# Metformin Antagonizes Cancer Cell Proliferation by Suppressing Mitochondrial-Dependent Biosynthesis

**DOI:** 10.1371/journal.pbio.1002309

**Published:** 2015-12-01

**Authors:** Takla Griss, Emma E. Vincent, Robert Egnatchik, Jocelyn Chen, Eric H. Ma, Brandon Faubert, Benoit Viollet, Ralph J. DeBerardinis, Russell G. Jones

**Affiliations:** 1 Goodman Cancer Research Centre, McGill University, Montreal, Quebec, Canada; 2 Department of Physiology, McGill University, Montreal, Quebec, Canada; 3 Children’s Medical Center Research Institute, University of Texas, Southwestern Medical Center at Dallas, Dallas, Texas, United States of America; 4 McDermott Center for Human Growth and Development, University of Texas, Southwestern Medical Center at Dallas, Dallas, Texas, United States of America; 5 Harold C. Simmons Comprehensive Cancer Center, University of Texas, Southwestern Medical Center at Dallas, Dallas, Texas, United States of America; 6 Inserm, U1016, Institut Cochin, Paris, France; 7 CNRS, UMR 8104, Paris, France; 8 Université Paris Descartes, Sorbonne Paris Cité, Paris, France; St. Jude Children's Research Hospital, UNITED STATES

## Abstract

Metformin is a biguanide widely prescribed to treat Type II diabetes that has gained interest as an antineoplastic agent. Recent work suggests that metformin directly antagonizes cancer cell growth through its actions on complex I of the mitochondrial electron transport chain (ETC). However, the mechanisms by which metformin arrests cancer cell proliferation remain poorly defined. Here we demonstrate that the metabolic checkpoint kinases AMP-activated protein kinase (AMPK) and LKB1 are not required for the antiproliferative effects of metformin. Rather, metformin inhibits cancer cell proliferation by suppressing mitochondrial-dependent biosynthetic activity. We show that in vitro metformin decreases the flow of glucose- and glutamine-derived metabolic intermediates into the Tricarboxylic Acid (TCA) cycle, leading to reduced citrate production and de novo lipid biosynthesis. Tumor cells lacking functional mitochondria maintain lipid biosynthesis in the presence of metformin via glutamine-dependent reductive carboxylation, and display reduced sensitivity to metformin-induced proliferative arrest. Our data indicate that metformin inhibits cancer cell proliferation by suppressing the production of mitochondrial-dependent metabolic intermediates required for cell growth, and that metabolic adaptations that bypass mitochondrial-dependent biosynthesis may provide a mechanism of tumor cell resistance to biguanide activity.

## Abstract 2

How does the antidiabetic drug metformin inhibit cancer? This metabolomic study shows that metformin blocks tumor cell proliferation independently of the classic metabolic checkpoints by suppressing mitochondrial-dependent biosynthesis.

## Author Summary

Cancer is a disease characterized by unregulated proliferation of transformed cells. To meet the increased biosynthetic demands of proliferation, biosynthetic building blocks required for cellular growth must be generated in large quantities. As cancer cells increase their anabolic metabolism to promote cell growth, there is significant interest in targeting these processes for cancer therapy. Metformin is a drug prescribed to treat Type II diabetes that has gained interest as an anti-tumor agent due to its suppressive effects on cancer cell proliferation. However, how metformin works to slow cancer cell growth has remained poorly understood. Here we show that metformin arrests cancer cell proliferation by starving mitochondria of the necessary metabolic intermediates required for anabolic metabolism in tumor cells. This results in reduced proliferation in part due to decreased synthesis of lipids used for membrane biosynthesis. We also show that some cancer cells use alternative metabolic pathways to synthesize lipids independently of mitochondrial metabolism, and that these cells are resistant to the antigrowth effects of metformin. Better understanding of mechanisms of metformin resistance will be crucial for metformin to be used as an effective anticancer agent.

## Introduction

Metformin is a member of the biguanide class of drugs used for the treatment of type II diabetes. Metformin directly inhibits complex I of the mitochondrial electron transport chain (ETC) [1,2], resulting in decreased complex I activity and oxidative phosphorylation (OXPHOS) in cells [3,4]. In diabetic patients, metformin primarily acts in the liver to inhibit gluconeogenesis [5–7], reducing hyperglycemia and the associated elevation in circulating insulin. Metformin functions in part by triggering an LKB1-dependent stress response in the liver, resulting in activation of the AMP-activated protein kinase (AMPK) energy sensor and reduced expression of gluconeogenic enzymes in hepatocytes [8]. However, recent epidemiological data has suggested that tumor progression is slowed in diabetic patients taking metformin versus patients on other antidiabetic treatments [9]. These results have driven considerable interest in investigating the use of metformin for cancer therapy.

Currently, there are two central models to explain the antiproliferative effects of metformin on cancer cells: 1) that metformin acts indirectly on tumor cell growth by lowering systemic insulin and insulin-like growth factor-1 (IGF-1) levels through inhibition of hepatic gluconeogenesis, thus suppressing the growth of insulin/IGF-1-dependent tumor cells; or 2) that metformin acts directly on complex I of tumor cells to reduce OXPHOS and other metabolic activities of tumor cells [10,11]. In support of the latter hypothesis, recent work has shown that metformin directly targets complex I of the ETC in cancer cells [1,2], and that complex I inhibition results in reduced cancer cell proliferation in vitro and in vivo [12]. However, the downstream effects of complex I inhibition and how they influence tumor proliferation remain unclear. Metformin-dependent effects on cellular bioenergetics can promote the activation of the metabolic checkpoint kinase AMPK [13–16], which has previously been linked to metformin action in tumor cells [14,17,18]. Metformin has also been proposed to suppress cell proliferation through inhibition of the mammalian target of rapamycin (mTOR) through AMPK-dependent and -independent pathways [19–21]. Given that metformin treatment reduces ETC activity and impacts glycolytic metabolism and lactate production, it remains possible that metformin may act by modulating metabolic pathways required for tumor cell growth and proliferation.

Here we have used metabolic profiling and stable isotope tracer analysis (SITA) to investigate the impact of metformin treatment on tumor cell metabolism. Our results indicate that metformin suppresses the flow of carbon into the Tricarboxylic Acid (TCA) cycle, which impacts pathways of mitochondrial-dependent biosynthesis including citrate-dependent de novo lipogenesis. We find that tumor cells with reduced oxidative mitochondrial function due to either mutations in the mitochondrial ETC or growth under hypoxia are resistant to metformin treatment; these cells continue to proliferate under metformin concentrations that normally suppress tumor cell growth. Together our data demonstrate that metformin can antagonize cell proliferation by suppressing TCA cycle-dependent production of metabolic intermediates required for tumor cell proliferation.

## Results

### The Effects of Metformin on Cancer Cell Proliferation and Metabolism Are Independent of Metabolic Checkpoints

**Fig 1:**
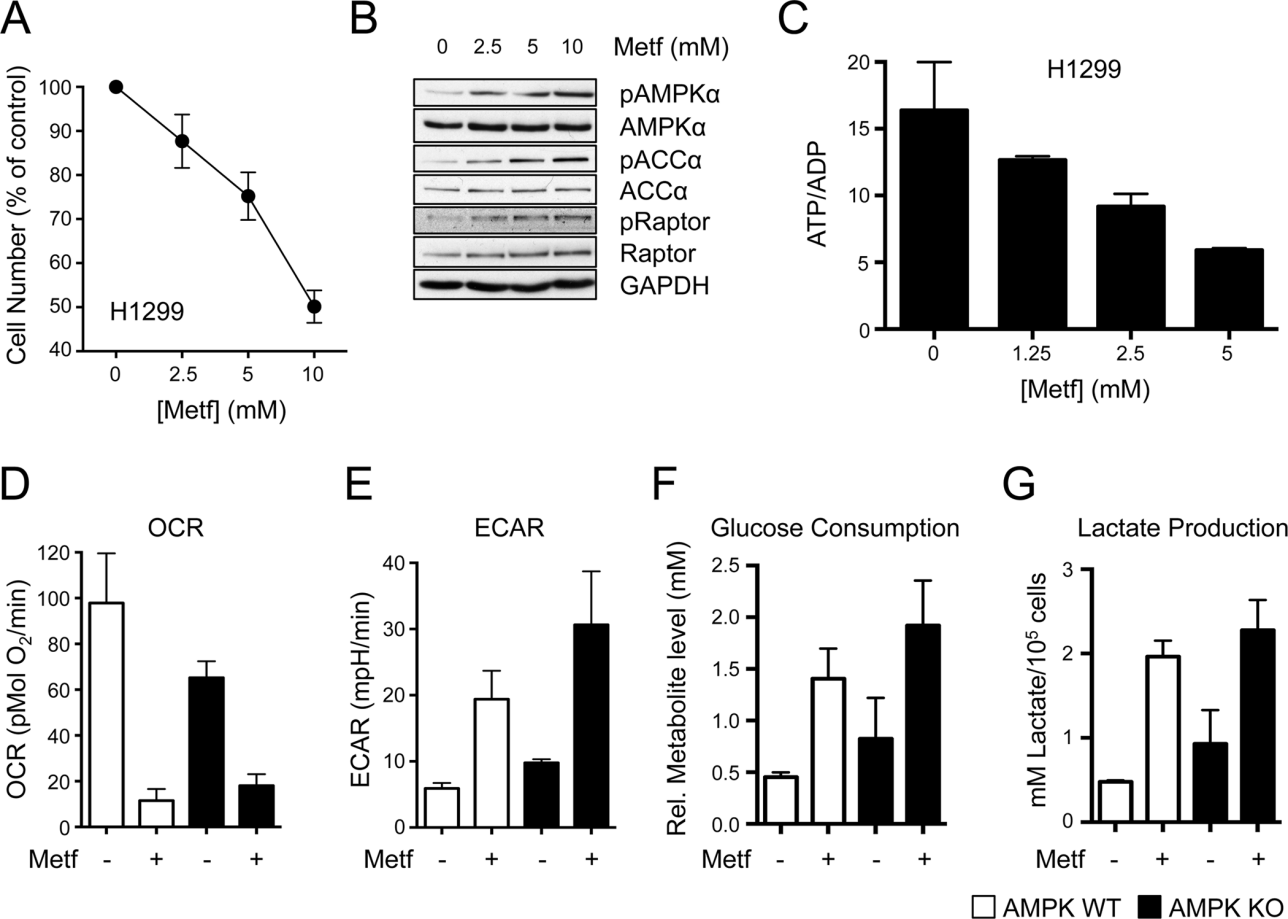
Metformin exerts AMPK-independent effects on cancer cell metabolism. **A.** Proliferation of H1299 NSCLC cells treated with the indicated concentrations of metformin for 72 h. Cell numbers are expressed relative to cell counts in control conditions (0 mM metformin). Each data point represents the mean ± standard error of the mean (SEM) for triplicate samples. **B.** Immunoblot of AMPK activation in metformin-treated H1299 cells. H1299 cells were treated for 1 h with various doses of metformin (from left to right: 0, 2.5, 5, and 10 mM), and cell lysates analyzed for AMPK (total and pT172), ACCα (total and pS79), and Raptor (total and pS792) levels. **C.** ATP:ADP ratio of H1299 cells cultured with varying doses of metformin for 14 h. Ratios are expressed relative to cells grown in complete growth medium. The data represents the mean ± standard deviation (SD) for triplicate samples. **D–G.** Metabolic characterization of metformin-treated mouse embryonic fibroblasts (MEFs). Wild type (WT) or AMPKα-deficient (knockout, KO) MEFs were cultured in the presence or absence of metformin. Shown are the O_2_ consumption rate (OCR) (**D**) and extracellular acidification rate (ECAR) (**E**) of cells cultured for 24 h in the presence or absence of 10 mM metformin. Glucose consumption (**F**) and lactate production (**G**) were assessed after 48 h of culture with metformin (5 mM). All data are normalized to cell number and represent the mean ± SEM for triplicate samples per condition. The data are representative of three independent experiments. Raw data for this figure can be found in S1 Data.

It is well established that metformin exerts antiproliferative effects on cancer cells in vitro [17,22,23]; however, the mechanisms of metformin action on tumor cells are poorly understood. We used H1299 non-small cell lung cancer (NSCLC) cells to better establish how metformin restricts cancer cell proliferation. Similar to other cancer cell types, H1299 cells display dose-dependent proliferative arrest in response to increasing metformin concentrations while maintaining cell viability (Fig 1A and S1A Fig). HCT116 colon cancer cells displayed a similar dose-dependent decrease in cell proliferation in response to increasing metformin concentrations (S1B Fig). AMPK signaling was intact in H1299 cells, as metformin treatment promoted increased phosphorylation of AMPKα (pT172) and increased phosphorylation of downstream AMPK targets ACCα (pS79) and Raptor (pS792) (Fig 1B and [24]). H1299 cells treated overnight with varying doses of metformin displayed a dose-dependent decrease in the ATP:ADP ratio (Fig 1C), consistent with the action of metformin as a mitochondrial complex I inhibitor [2–4]. Cellular ATP levels decreased with increasing doses of metformin treatment, along with increases in ADP and AMP levels in metformin-treated cells (S1C Fig).

We next examined the bioenergetic profiles of metformin-treated cells by Seahorse assay, which measures the extracellular acidification rate (ECAR, a measure of extracellular pH that correlates with glycolysis) and O_2_ consumption rate (OCR, a measure of OXPHOS) of cells in real time [25]. H1299 cells displayed decreased OCR and increased ECAR after acute metformin treatment (S1D and S1E Fig), indicating reduced OXPHOS and a metabolic shift towards glycolysis. Extended metformin treatment (>24 h) promoted increased glucose consumption and lactate production relative to untreated cells (S1F and S1G Fig). The energy sensor AMPK has previously been linked to the antiproliferative and metabolic effects of metformin [14,17,22], so we examined the effects of metformin on the metabolism of mouse embryonic fibroblasts (MEFs) lacking the α catalytic subunits of AMPK (*α1* ^*-/-*^ *α2* ^*-/-*^, knockout [KO]) [26]. Metformin treatment reduced OXPHOS (Fig 1D) and increased the ECAR (Fig 1E) of MEFs regardless of AMPK expression. Metformin also increased glucose consumption (Fig 1F) and lactate production (Fig 1G) in both control and AMPKα-deficient MEFs.

**Fig 2:**
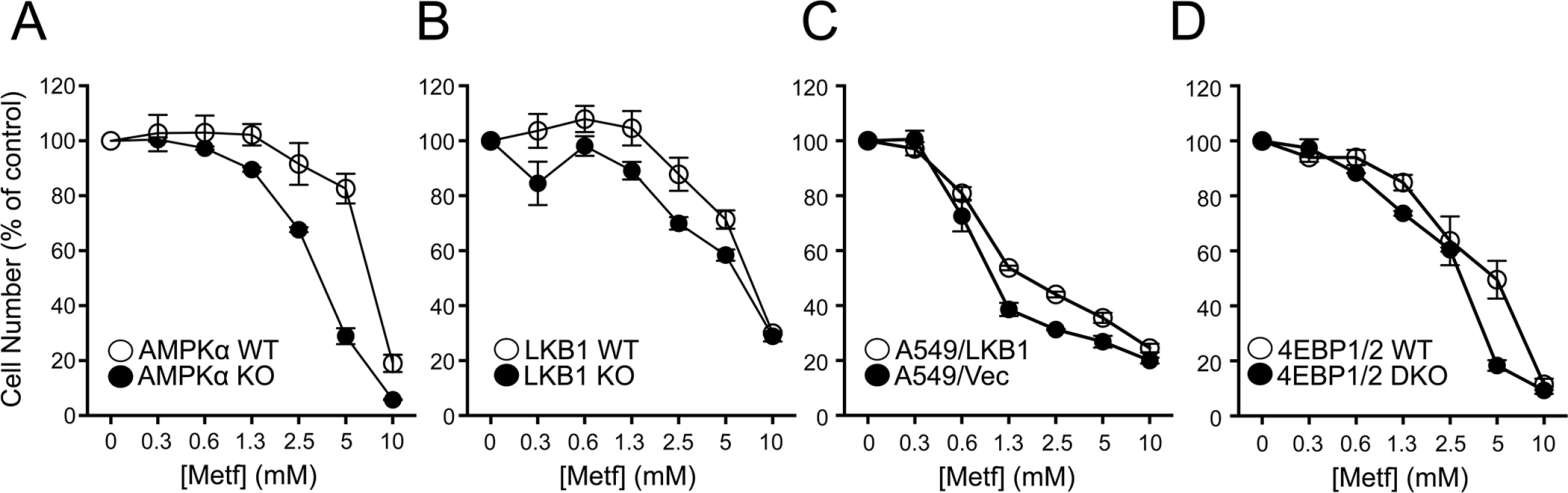
Metformin suppresses cancer cell proliferation independent of metabolic checkpoints. Cells were cultured with the indicated concentrations of metformin, and cell number was determined by crystal violet incorporation after 72 h of culture. Cell number is expressed relative to control cell number (no metformin) at 72 h. Shown are cell numbers for control (WT) and AMPKα-deficient (KO) MEFs (**A**), control (WT) and LKB1-deficient (KO) MEFs (**B**), A549 cells expressing empty vector (A549/Vec) or re-expressing LKB1 (A549/LKB1) (**C**), and control (WT) or 4EBP1/2-deficient (double knockout, DKO) MEFs (**D**) in response to metformin treatment. Each data point represents the mean ± SEM for triplicate samples. Raw data for this figure can be found in S2 Data.

We next examined the impact of metformin on the proliferation of cells lacking specific metabolic checkpoints. We first examined the impact of metformin treatment on the proliferation of MEFs lacking AMPKα1 and α2, which eliminates all AMPK catalytic activity [27]. Proliferation was inhibited in both AMPK WT and KO MEFs, with AMPK KO MEFs displaying increased sensitivity to metformin treatment (Fig 2A). MEFs lacking LKB1, which is required for AMPK activation in response to biguanide treatment [28], displayed similar reductions in cell proliferation in response to metformin as control cells (Fig 2B). A549 lung carcinoma cells lacking LKB1 displayed a similar dose-dependent suppression of cell proliferation in response to metformin treatment as A549 cells expressing LKB1 (Fig 2C). Metformin is also a potent inhibitor of mTORC1 activity [19,20,29]. mTORC1 promotes cell proliferation by phosphorylating and inhibiting the eIF4E binding proteins 4EBP-1 and -2, and cells lacking 4EBP1/2 continue to proliferate even when mTORC1 activity is inhibited [30]. Thus, we examined the antiproliferative effects of metformin on 4EBP-1/2-deficient MEFs. Both control and 4EBP1/2 KO MEFs displayed similar reductions in cell proliferation in the presence of metformin (Fig 2D), suggesting that the cytostatic effects of metformin do not depend on mTORC1-dependent inhibition of 4EBPs. Treatment with the biguanide phenformin induced similar antiproliferative effects as metformin on LKB1-deficient A549 cells (S2A Fig) and 4EBP1/2-deficient MEFs (S2B Fig), although at lower drug concentrations.

### Metformin Inhibits Glucose and Glutamine Utilization in Mitochondria

**Fig 3:**
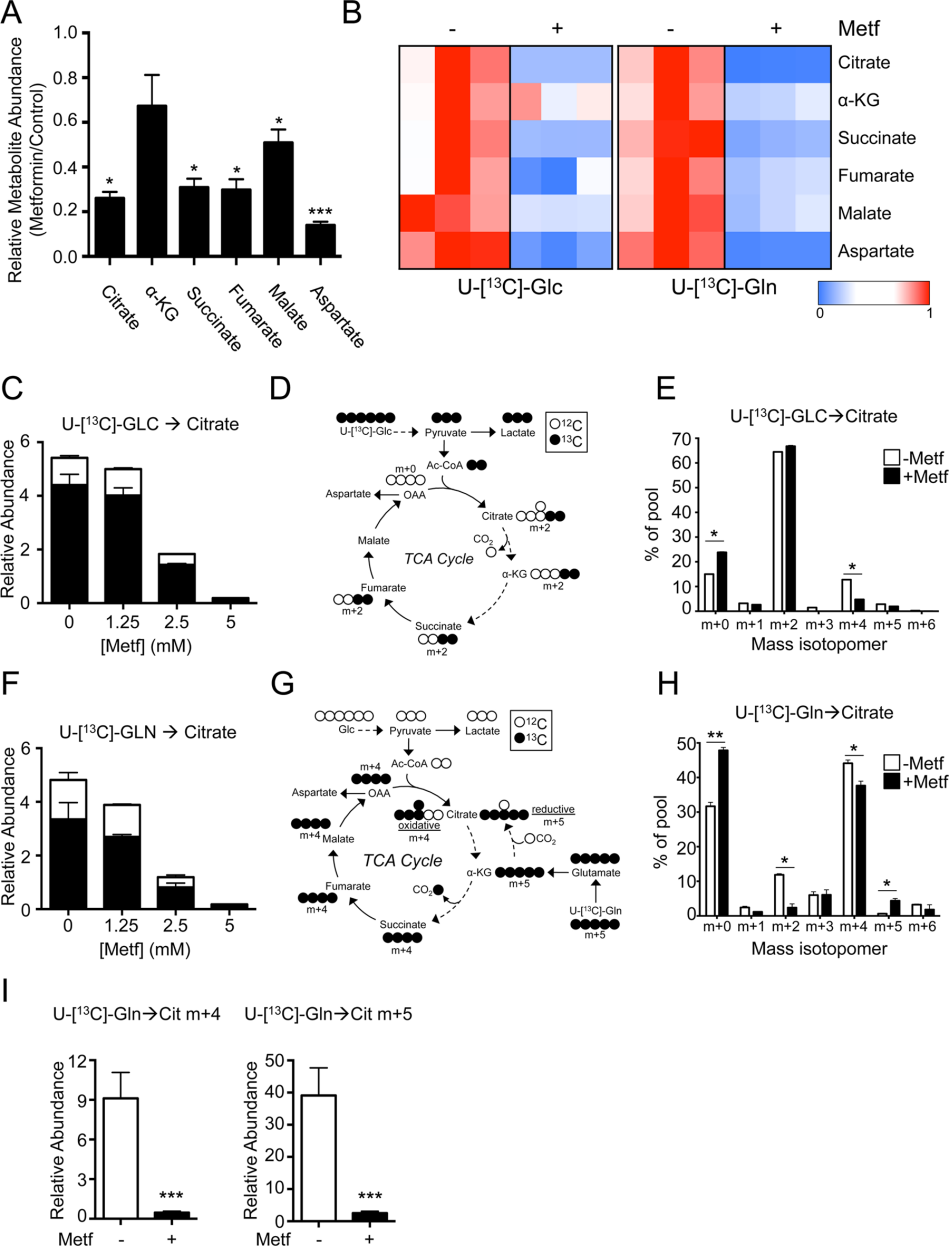
Metformin suppresses glucose- and glutamine-dependent TCA cycle activity. **A.** Relative abundance of TCA metabolites in metformin-treated H1299 cells. Cells were treated with or without metformin (5 mM) for 8 h, and TCA cycle metabolites determined by gas chromatography-mass spectrometry (GC-MS). Data are expressed as the ratio of metabolite levels in metformin-treated cells relative to cells cultured without metformin. Data shown is normalized to cell number. The data represent the mean ± SEM for triplicate samples. **B.** Heat map of relative metabolite abundances in metformin-treated H1299 cells. H1299 cells were treated with (+) or without (−) 5 mM metformin for 6 h, followed by culture with U-[^13^C]-glucose or U-[^13^C]-glutamine for an additional 2 h. Shown is the relative abundance of U-[^13^C]-glucose-derived (left panel) or U-[^13^C]-glutamine-derived (right panel) TCA cycle metabolites under each culture condition. Data are expressed relative to the ^13^C metabolite abundance in H1299 cells cultured under control conditions (no metformin). **C.** Relative abundance of glucose-derived citrate in metformin-treated H1299 cells. Cells were treated for 24 h with the indicated doses of metformin followed by incubation with U-[^13^C]-glucose for 2 h. The abundance of unlabeled (^12^C, white) and U-[^13^C]-glucose-labeled (^13^C, black) citrate was determined by GC-MS. Data are normalized to cell number. **D.** Schematic of U-[^13^C]-glucose labeling in the TCA cycle. Input of fully-labeled Ac-CoA (m + 2) results in the generation of m + 2-labeled metabolites on the first turn of the TCA cycle, and m + 4-labeled metabolites on the second turn. **E.** Distribution of U-[^13^C]-glucose-derived isotopomers of citrate in H1299 cells cultured with or without metformin as in (**B**). The data represent the mean ± SEM for triplicate samples. **F.** Relative abundance of glutamine-derived citrate in metformin-treated H1299 cells. Cells were treated as in (**B**), and the abundance of unlabeled (^12^C, white) and U-[^13^C]-glutamine-labeled (^13^C, black) citrate was determined by GC-MS. Data are normalized to cell number. **G.** Schematic of U-[^13^C]-glutamine labeling in the TCA cycle. Anaplerotic U-[^13^C]-glutamine flux into the TCA cycle follows clockwise flow resulting in m + 4 labeling during the first round of the TCA cycle. Reductive carboxylation of α-KG results in m + 5 labeling in citrate. **H.** Distribution of U-[^13^C]-glutamine-derived isotopomers of citrate in H1299 cells cultured with or without metformin as in (**B**). The data represent the mean ± SEM for triplicate samples. **I.** Relative abundance of citrate produced via oxidative (m + 4) and reductive (m + 5) pathways in H1299 cells treated with (+) or without (−) metformin. Cells were treated as in (**B**), and the abundance of U-[^13^C]-glutamine-labeled m+4 and m+5 citrate was determined by GC-MS. *, *p* < 0.05; **, *p* < 0.01; ***, *p* < 0.001. Raw data for this figure can be found in S3 Data.

Given the suppressive effect of metformin on OXPHOS, we next investigated the impact of metformin treatment on pathways of oxidative metabolism. A general screen of TCA metabolites in H1299 cells revealed that metformin treatment significantly lowered all metabolites of the TCA cycle except α-ketoglutarate (α-KG) (Fig 3A). Using SITA with U-[^13^C]-glucose or U-[^13^C]-glutamine, we found that metformin treatment reduced the incorporation of glucose- and glutamine-dependent carbon into TCA cycle metabolite pools (Fig 3B). We observed a dose-dependent reduction in the total abundance of ^13^C-glucose-derived citrate under metformin treatment (Fig 3C). Examination of ^13^C isotopomer distribution in cells incubated with U-[^13^C]-glucose (schematic in Fig 3D) revealed an increase in unlabeled citrate (m + 0) in metformin-treated cells (Fig 3E). This could result from either reduced pyruvate entry into the TCA cycle due to increased lactate production (Fig 1G) or decreased cycling of citrate through the TCA cycle, as distribution of m + 4-labeled citrate (generated from two cycles of m + 2 acetyl-CoA entering the TCA cycle) was significantly reduced in metformin-treated cells (Fig 3E). Similar reductions in ^13^C-labeling from glucose were also observed in the TCA cycle metabolites succinate and fumarate in metformin-treated cells (S3A Fig).

Glutamine contributes to anaplerotic flux into the TCA cycle via deamination and transamination reactions to generate α-KG. Metformin treatment of H1299 cells reduced the total abundance of citrate, succinate, and fumarate derived from U-[^13^C]-glutamine (Fig 3F and S2B Fig). Oxidative metabolism of α-KG (m + 5 labeled when derived from U-[^13^C]-glutamine) results in citrate labeled as m + 4, while reductive metabolism of α-KG by isocitrate dehydrogenase (IDH) generates m + 5 citrate (Fig 3G). Consistent with previous observations [31,32], we observed an increased proportion of m + 5 citrate in the total citrate pool following metformin treatment of H1299 cells (Fig 3H), suggesting a shift in α-KG metabolism towards reductive carboxylation. This shift in α-KG metabolism has previously been argued as an adaptive response to metformin treatment in certain tumor types [31]. However, the total abundance of m + 5 citrate was significantly reduced in metformin-treated cells (~5% of that observed in control treated cells) despite an overall metabolic shift towards reductive carboxylation of α-KG (Fig 3I). Lastly, the decrease in citrate abundance under metformin treatment was independent of AMPK expression (S3C Fig).

### Metformin Suppresses De Novo Lipid Synthesis in Tumor Cells

**Fig 4:**
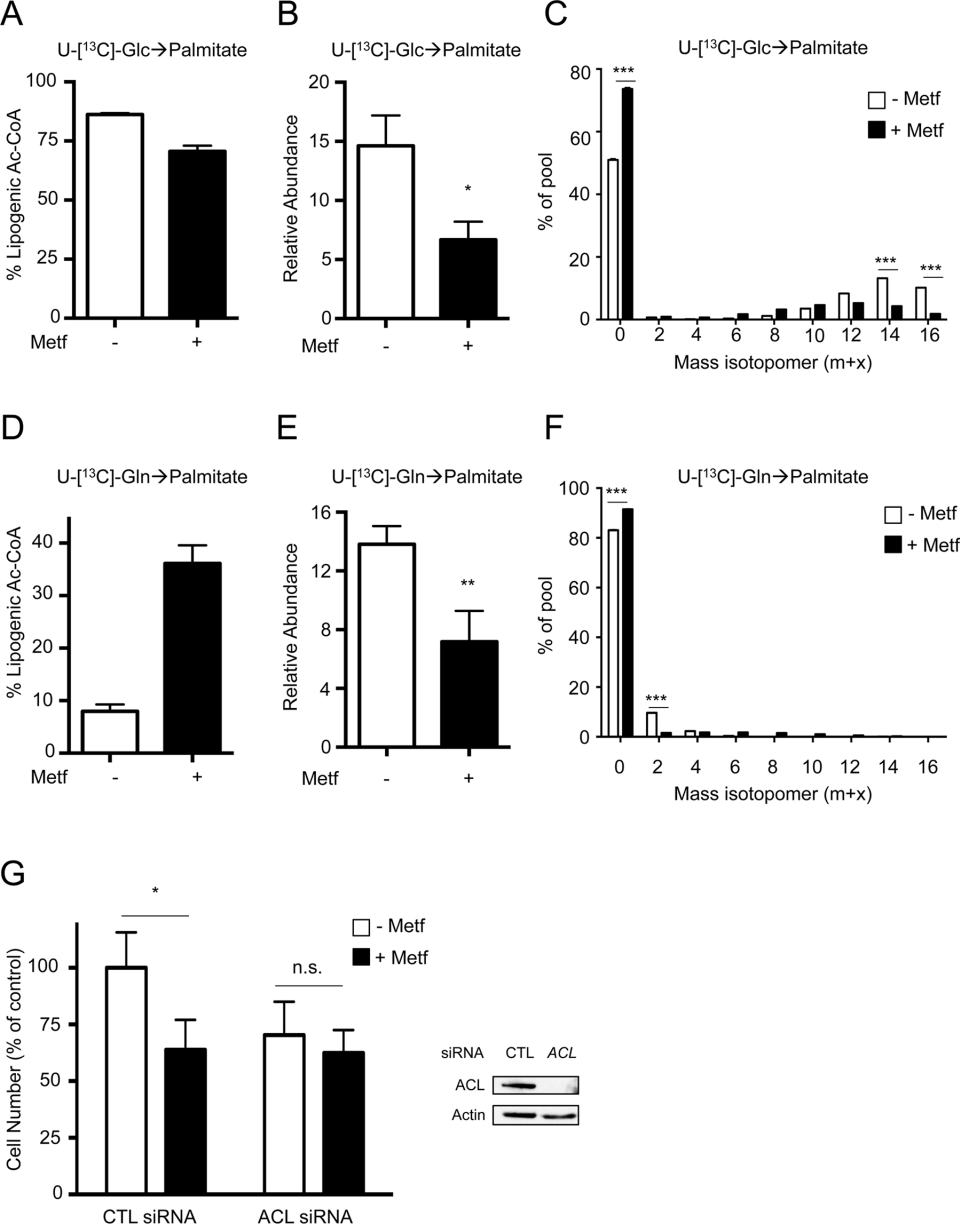
Metformin inhibits de novo palmitate synthesis in cancer cells. A–F. Palmitate biosynthesis in metformin-treated H1299 cells. Cells were cultured for 48 h with (+) or without (−) 5 mM metformin, followed by an additional 24 h of culture with U-[^13^C]-glucose or U-[^13^C]-glutamine. Relative lipogenic acetyl-CoA derived from labeled glucose (**A**) or labeled glutamine (**D**). U-[^13^C]-glucose-derived (**B**) or U-[^13^C]-glutamine-derived (**E**) palmitate was determined by GC-MS. Data are normalized to cell numbers. **C, F.** Mass isotopomer distribution of U-[^13^C]-glucose-derived (**C**) or U-[^13^C]-glutamine-derived (**F**) palmitate. **G.** Proliferation of H1299 NSCLC cells expressing control (CTL) or ATP citrate lyase (ACL)-specific siRNAs following culture for 72 h with (+) or without (−) 5 mM metformin. Cell numbers are expressed relative to cell counts in control conditions (0 mM metformin), and data represent the mean ± SEM for each condition (*n* = 20). Levels of ACL knockdown in H1299 cells were determined by immunoblot. *, *p* < 0.05; **, *p* < 0.01. Raw data for this figure can be found in S4 Data.

Citrate is a key intermediate for the production of cytosolic acetyl-CoA, which is a substrate for de novo lipid biosynthesis. Given the reduced citrate production induced by metformin, we assessed the impact of metformin treatment on de novo fatty acid (FA) synthesis. The relative abundance of palmitate, one of the most abundant fatty acids in H1299 cells, was reduced following metformin treatment (S3A Fig). Isotope tracer analysis using U-[^13^C]-glucose revealed a decrease in the lipogenic acetyl-CoA derived from glucose (Fig 4A). Consequently, we observed a decrease in the relative abundance of ^13^C-labeled palmitate following metformin treatment (Fig 4B). Moreover, we observed an increase in un-labeled (m + 0) palmitate upon metformin treatment (Fig 4C). Conversely, levels of U-[^13^C]-glutamine-derived lipogenic acetyl-CoA pools increased upon metformin treatment (Fig 4D). Yet, the relative abundance of glutamine-derived palmitate was decreased (Fig 4E) due to lowered citrate abundance (Fig 3F). Consistent with this, there was an increase in unlabeled (m + 0) palmitate upon metformin treatment combined with U-[^13^C]-glutamine (Fig 4F). Metformin treatment lowered U-[^13^C]-glucose- and -glutamine-derived lipogenic acetyl-CoA pools in both WT and AMPKα-deficient MEFs, indicating that the effects of metformin on de novo lipogenesis were AMPK-independent (S3B and S3C Fig). Furthermore, metformin reduced both lipogenic acetyl-CoA production and palmitate biosynthesis in the presence of the β-oxidation inhibitor etomoxir (S3D Fig), suggesting that reduced lipid synthesis, rather than increased beta-oxidation, was the cause of reduced palmitate abundance in metformin-treated cells.

ATP citrate lyase (ACL) converts cytosolic citrate into acetyl-CoA, and is a rate-limiting step for FA synthesis in tumor cells [33]. Metformin reduced the proliferation of H1299 cells to the same extent as cells in which ACL has been silenced using siRNA (Fig 4G), consistent with reduced citrate-dependent lipid biosynthesis as a mechanism for metformin action.

### Metformin-Dependent Inhibition of Lipid Biosynthesis Requires Functional Mitochondria

Recent reports have linked inhibition of complex I of the mitochondrial ETC to the antiproliferative effects of metformin in cells with functional mitochondria [1,12]. However, tumor cells grown under hypoxia or harboring defects in mitochondrial metabolism are able to generate biosynthetic intermediates for cell growth via glutamine-dependent reductive carboxylation [32,34,35]. Our data argue that cells with defective ETC activity may be refractive to metformin treatment. To test this hypothesis, we examined the impact of metformin on cellular biosynthesis and growth of isogenic 143B human osteosarcoma cells with intact (143B*wt*) or defective (143B*cytb*) complex III activity [32,36,37]. Cells with nonfunctional complex III displayed decreased OCR (S4A Fig) and a slight increase in ECAR (S4B Fig), which was unaffected by metformin treatment, confirming defective OXPHOS in these cells. While mutant 143B*cytb* cells displayed lower overall levels of TCA cycle intermediates compared to control 143B*wt* cells, metformin treatment did not affect TCA metabolite levels (with the exception of succinate) in cells lacking functional ETC activity (S5C Fig).

**Fig 5:**
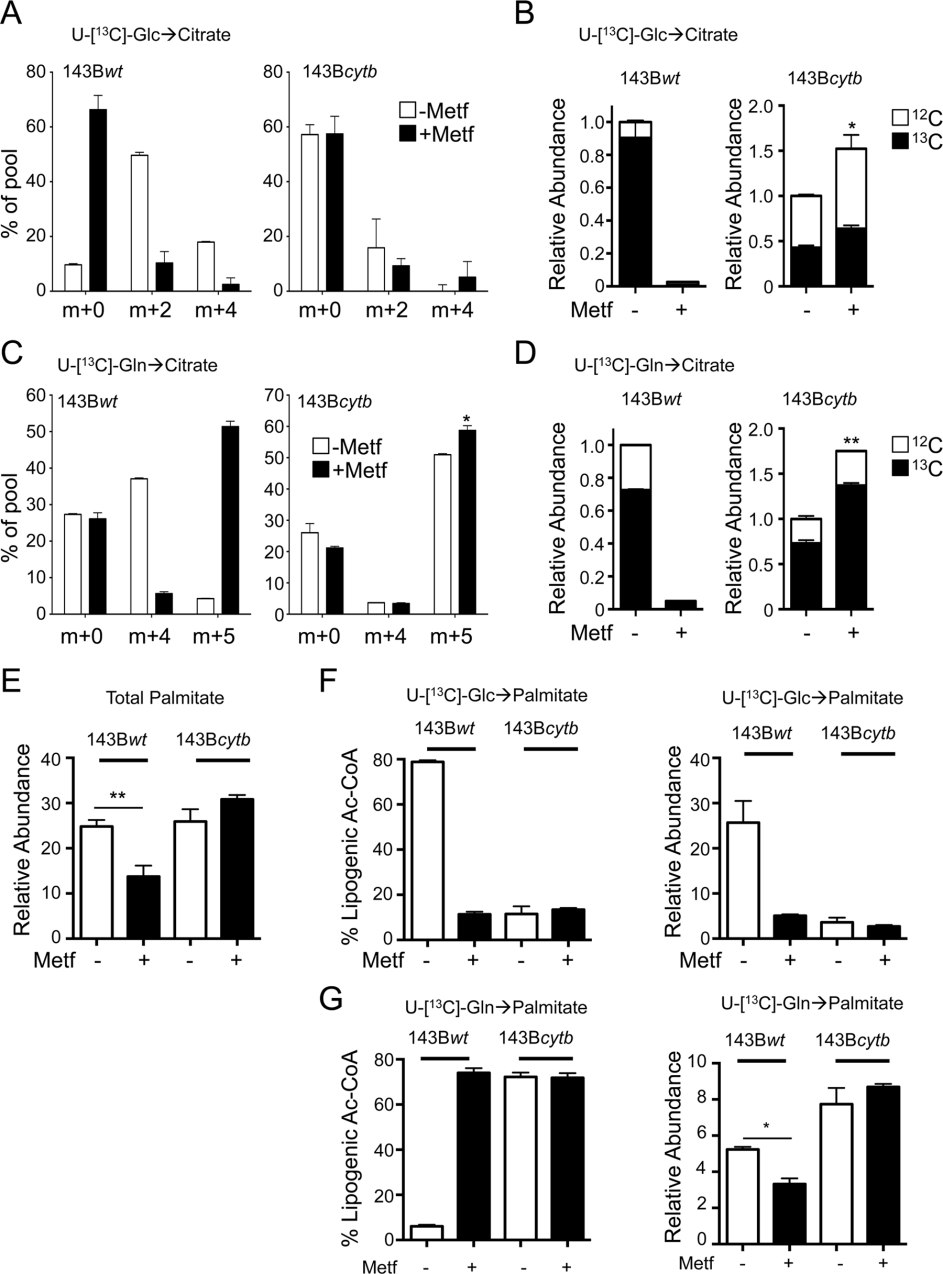
Metformin requires functional mitochondrial electron transport to suppress de novo lipogenesis. A–B. Abundance and distribution of U-[^13^C]-glucose-derived citrate in metformin-treated 143B osteosarcoma cells. 143B*wt* and 143B*cytb* cells were cultured with (+) or without (−) metformin (10 mM) for 12 h, and intracellular metabolites extracted and analyzed by GC-MS. U-[^13^C]-glucose was added for the final 6 h of culture. Shown is the isotopomer distribution (**A**) and relative abundance (**B**) of U-[^13^C]-glucose-derived citrate in 143B*wt* and 143B*cytb* cells treated as indicated. Data are normalized to cell number and are presented as mean ± SEM for each condition (*n* = 3), and are representative of two independent experiments. **C–D.** Abundance and distribution of U-[^13^C]-glutamine-derived citrate in metformin-treated 143B cells. 143B Cells were treated as in (**A**), with U-[^13^C]-glutamine added for the final 6 h of culture. Isotopomer distribution (**C**) and relative abundance (**D**) of U-[^13^C]-glutamine-derived citrate in 143B*wt* and 143B*cytb* cells is shown. Data are normalized to cell number. **E**. Relative palmitate abundance in 143B*wt* and 143B*cytb* cells cultured with (+) or without (−) metformin (10 mM) for 72 h. Data are normalized to cell number and presented as mean ± SEM for each condition (*n* = 3). **F–G**. Relative abundance of U-[^13^C]-glucose-derived (**F**) and U-[^13^C]-glutamine-derived (**G**) lipogenic acetyl-CoA and palmitate in 143B*wt* and 143B*cytb* cells cultured in the presence (+) or absence (−) of metformin (10 mM) for 72 h. Cells were cultured for 72 h, with U-[^13^C]-glucose or U-[^13^C]-glutamine added for the final 24 h of culture. Data are normalized to cell number, are presented as mean ± SEM for triplicate samples, and are representative of three independent experiments. *, *p* < 0.05; **, *p* < 0.01. Raw data for this figure can be found in S5 Data.

Isotope tracer analysis of 143B cells using U-[^13^C]-glucose indicated that metformin selectively suppressed citrate biosynthesis in cells with functional ETC activity (Fig 5A). Metformin treatment increased the percentage of unlabeled (m + 0) citrate in 143B*wt* but not 143B*cytb* cells, while concurrently decreasing levels of m + 2 and m + 4 citrate (Fig 5A). This resulted in a selective depletion of ^13^C-glucose-derived citrate in 143B*wt* cells (Fig 5B). As reported previously [32], metformin could promote reductive glutamine metabolism in 143B*wt* cells (generation of m + 5 citrate from U-[^13^C]-glutamine), while 143B*cytb* cells displayed reductive carboxylation even in the absence of metformin (Fig 5C). While the abundance of U-[^13^C]-glutamine-derived citrate in 143B*wt* cells was significantly impacted by metformin, 143B*cytb* cells without functional ETC activity continued to generate citrate from glutamine and actually displayed an increase in ^13^C-glutamine-derived citrate when cultured with metformin (Fig 5D).

We next assessed whether metformin could suppress FA biosynthesis in cells lacking functional ETC activity. Total palmitate levels in 143B*wt* cells were significantly decreased by metformin treatment, while palmitate levels in 143B*cytb* cells were unaffected (Fig 5E). Isotope tracing experiments with U-[^13^C]-glucose revealed that palmitate is largely derived from glucose in 143B*wt* cells, and that metformin significantly reduced the production of glucose-derived lipogenic acetyl-CoA and palmitate in 143B*wt* cells (Fig 5F). 143B*wt* cells were unable to compensate by increasing the level of U-[^13^C]-glutamine-derived palmitate despite increasing the U-[^13^C]-glutamine-derived lipogenic acetyl-CoA (Fig 5G), by switching to reductive glutamine metabolism in response to metformin (Fig 5C). In contrast, 143B*cytb* derived very little lipogenic acetyl-CoA and palmitate from U-[^13^C]-glucose (Fig 5F). Rather, levels of U-[^13^C]-glutamine-derived lipogenic acetyl-CoA and palmitate were higher in 143B*cytb* cells compared to 143B*wt* cells, and both total abundances and isotopomer distribution of U-[^13^C]-glutamine-derived palmitate were unaffected by metformin treatment (Fig 5G and S5D Fig).

### Cells That Bypass Mitochondrial-Dependent Biosynthetic Pathways Are Resistant to the Antiproliferative Effects of Metformin

**Fig 6:**
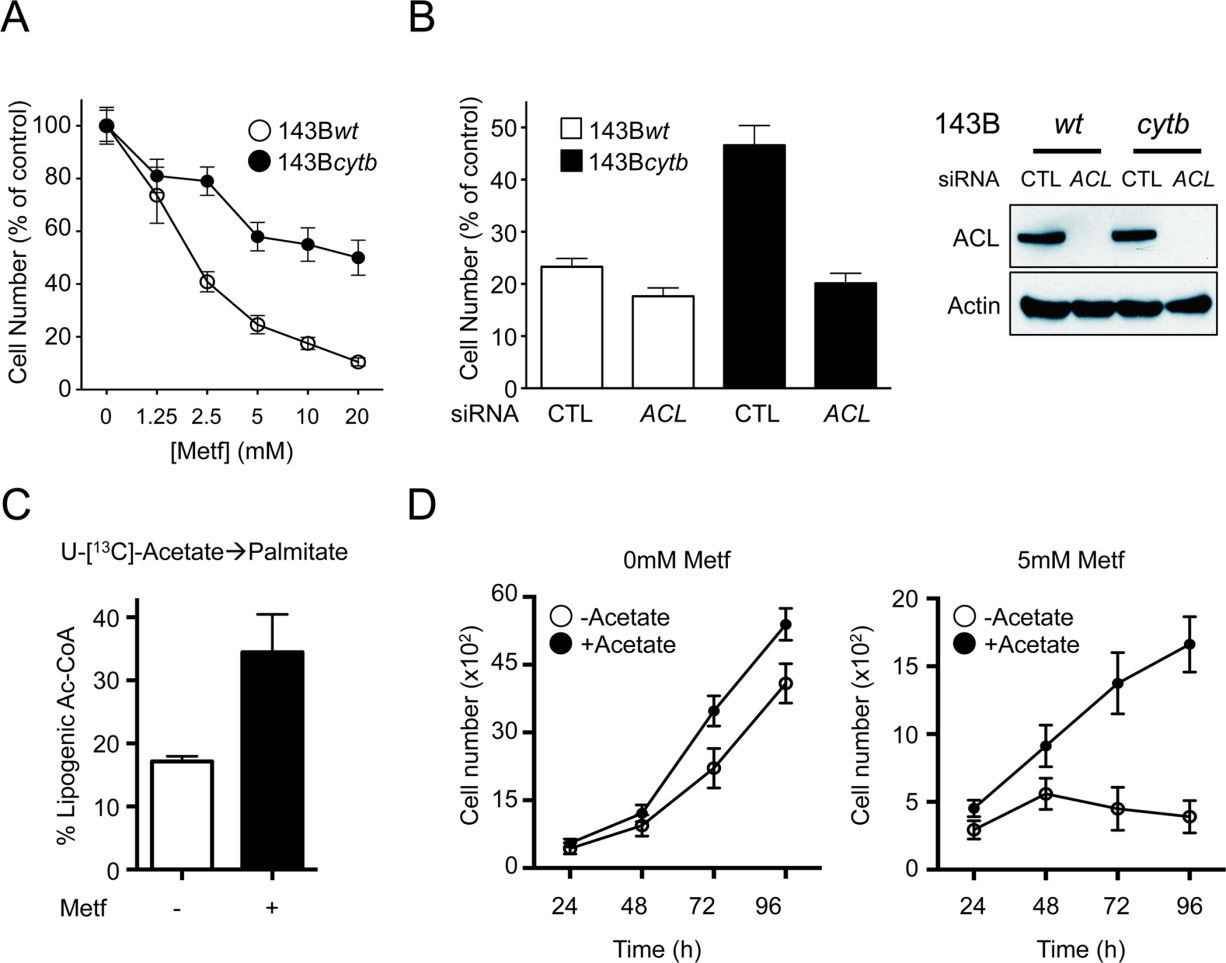
Cancer cells with defective ETC activity display resistance to the antiproliferative effects of metformin. **A.** Proliferation of 143B*wt* and 143B*cytb* cells cultured in medium containing the indicated doses of metformin. Cell numbers were determined after 72 h of treatment, and are expressed relative to cell counts in control conditions (0 mM metformin). Each data point represents the mean ± SEM for each condition (*n* = 10). **B.** Proliferation of 143B*wt* and 143B*cytb* cells treated with control siRNA (CTL) or ACL-specific siRNA. Cell counts were determined after 48 h of culture in medium containing 10 mM metformin and expressed relative to cell counts in control conditions. The data represent the mean ± SEM for each condition (*n* = 10) and are representative of three independent experiments. **C.** U-[^13^C]-acetate-derived lipogenic acetyl-CoA in 143B*wt* cells with or without 5 mM metformin treatment for 72 h. **D.** Proliferation of 143B*wt* cells cultured in the absence or presence of 5 mM metformin under control (white) or acetate supplementation (black, 5 mM) conditions. Growth curves over time are shown. Each data point represents the mean ± SD for each condition (*n* = 10), and is representative of three independent experiments. Raw data for this figure can be found in S6 Data.

Given that 143B*cytb* cells with defective mitochondrial ETC activity were insensitive to the effects of metformin on citrate production and palmitate biosynthesis, we next investigated the proliferation of these cells in the presence of metformin. Metformin treatment induced a dose-dependent decrease in proliferation of 143B*wt* cells (Fig 6A), correlating with decreased citrate production and FA biosynthesis in these cells (Fig 5). Conversely, 143B*cytb* cells displayed greater resistance to the antiproliferative effects of metformin treatment at all concentrations tested (Fig 6A), while not affecting cell viability (S5A Fig). Given that glutamine-dependent FA biosynthesis remained intact in 143B*cytb* cells (Fig 5G), we assessed the impact of silencing ACL on the proliferation of 143B*cytb* cells (Fig 6B). While 143B*cytb* cells maintained higher levels of proliferation in response to metformin relative to 143B*wt* cells, silencing ACL attenuated the growth advantage of 143B*cytb* cells, lowering their proliferation to that seen in metformin-treated 143B*wt* cells (Fig 6B and S5B Fig). We observed that silencing ACL did not result in a buildup of citrate pools in the mitochondria. Rather, glutamine-derived citrate was still lowered under metformin treatment in ACL-silenced 143B*wt* cells (S5C Fig). We also observed a lower baseline level of citrate when ACL is silenced, which is consistent with a slower proliferative rate for these cells under control conditions (S6C Fig).

The reduction of mitochondrial-dependent lipogenesis by metformin correlates with its suppressive effect on proliferation. To assess the importance of de novo lipogenesis to metformin-dependent proliferative arrest, we provided metformin-treated cells with acetate, which can function as an external lipogenic substrate for cancer cells [38]. U-[^13^C]-Acetate tracing experiments confirmed that acetate could contribute to the lipogenic acetyl-CoA pool in metformin-treated cells (Fig 6C). 143B*wt* tumor cells supplemented with acetate displayed a slight increase in cell proliferation under regular growth conditions (Fig 6D). Importantly, acetate supplementation resulted in sustained proliferation of 143B*wt* cells when cultured in the presence of metformin (Fig 6D).

**Fig 7:**
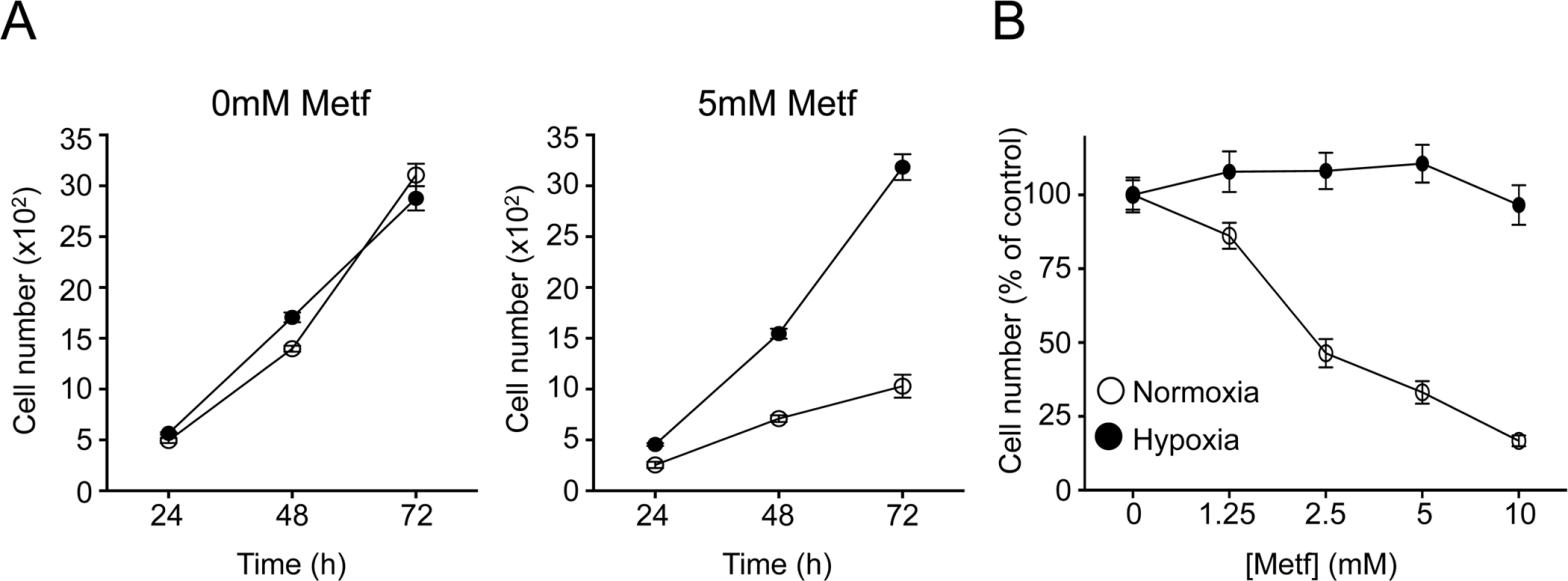
Hypoxia results in adaptive resistance to the anti-proliferative effects of metformin. **A**. Proliferation of 143B*wt* cells cultured in the absence or presence of 5 mM metformin under normoxic (white) or hypoxic (black, 1% O_2_) conditions. Growth curves over time are indicated. **B.** Proliferation of 143B*wt* cells cultured under normoxia (white) or hypoxia (black, 1% O_2_) in medium containing the indicated doses of metformin. Cell numbers were determined after 72 h of treatment, and are expressed relative to cell counts in control conditions. Each data point represents the mean ± SEM for each condition (*n* = 10), and is representative of three independent experiments. Raw data for this figure can be found in S7 Data.

Tumor cells growing under hypoxia use reductive glutamine metabolism as a means to bypass mitochondrial-dependent lipogenesis [34,35]. Thus, we assessed the growth rate of cancer cells under both hypoxia and metformin treatment. Metformin treatment reduced 143B*wt* cell proliferation under normoxic conditions (~20% O_2_), but had no impact on cell proliferation when cells were cultured under hypoxic conditions (1% O_2_) (Fig 7A), even over a broad range of metformin concentrations (Fig 7B). Together, these data suggest that metformin can antagonize cell proliferation by suppressing mitochondrial-dependent lipogenesis, and that cells capable of bypassing this mitochondrial-dependent biosynthetic pathway are resistant to the antiproliferative properties of biguanides.

## Discussion

Recent work has confirmed that biguanides such as metformin can exert cell autonomous, dose-dependent effects on tumor cell proliferation [12,39]. While inhibition of complex I activity is required for the antitumor effects of metformin [1,12], the mechanism(s) by which metformin affects proliferation downstream of complex I inhibition has remained unclear. One hallmark of proliferating cancer cells is the reprogramming of cellular metabolism to direct resources towards cellular biosynthetic pathways to support anabolic growth [40]. Our results here indicate that metformin can limit tumor cell proliferation in vitro by starving them of the biosynthetic intermediates required for cell growth. Mitochondrial-derived TCA cycle intermediates such as citrate are key substrates required for the production of biomass [41,42]. We show that treatment of cancer cells with metformin reduces glucose- and glutamine-dependent anaplerotic flux into the TCA cycle, leading to depletion of citrate-derived acetyl-CoA required for de novo lipid biosynthesis. Interestingly, the metabolic effects of metformin are independent of the metabolic checkpoint kinases LKB1 and AMPK. However, tumor cells capable of mitochondrial-independent citrate production—including cells growing under hypoxia or with impaired ETC activity—are insensitive to the antiproliferative effects of metformin. Together our data argue for a “substrate limitation” model of metformin action, by which metformin restricts biosynthetic capacity by reducing the production of TCA cycle intermediates required for cell growth.

Our data indicate that metformin exerts dose-dependent cytostatic effects on cancer cell proliferation without major alterations in cell viability. This is likely due to the metabolic shift to glycolysis triggered by metformin-dependent suppression of complex I [1,12]. In fact, blocking glycolysis in metformin-treated cells can lead to metabolic crisis and reduced viability of tumor cells [43]. It has been argued that activation of metabolic checkpoints by LKB1 and AMPK are necessary for the adaptive metabolic response of tumor cells to metformin [14,17,22]. Activation of AMPK downstream of metformin treatment can suppress mTORC1-dependent mRNA translation in tumor cells [20], and mTORC1 can exert global effects on cellular metabolism [44,45]. However, we find that AMPK-deficient cells are capable of inducing the metabolic switch to glycolysis promoted by metformin treatment, and that cells with defective LKB1-AMPK signaling remain sensitive to the cytostatic effects of metformin. Moreover, cells lacking the translation inhibitors 4E-BP1/2 also display reduced proliferation in response to metformin treatment. Deletion of 4E-BP1/2 normally helps maintain mTORC1-dependent cell proliferation under growth-restrictive conditions [30], yet these cells enter growth arrest despite maintenance of eIF4E-dependent translation. While activation of these checkpoints may be important for surviving the energetic stress associated with chronic metformin treatment [28,46–48], our data indicate that metformin is capable of stimulating metabolic changes and growth arrest independent of AMPK.

Cancer cells rely on the input of both glucose- and glutamine-derived carbon to maintain mitochondrial respiration and ATP production. However, these nutrients also contribute to biomass generation and can thus be limiting factors for proliferating cells [49]. Using ^13^C tracer analysis, we found that metformin greatly reduced the anaplerotic flux of both glucose and glutamine into the TCA cycle, leading to a significant reduction in TCA cycle metabolite abundance. In particular, citrate production from U-[^13^C]-glucose and U-[^13^C]-glutamine was highly impacted by metformin treatment, reduced by 75% and >90%, respectively. This reduced citrate production had a direct impact on the ability of metformin-treated cells to direct glucose and glutamine carbon into lipid biosynthesis, as measured by a reduction in glucose- and glutamine-derived lipogenic acetyl-coA pools and reduced ^13^C labeling in palmitate under metformin conditions. Targeting ACL, a rate-limiting step for converting cytosolic citrate to acetyl-CoA for fatty acid biosynthesis [50], inhibited cancer cell proliferation to a similar extent as that induced by metformin treatment. Alternatively, we show that providing tumor cells with an alternate lipogenic substrate such as acetate can help overcome the antiproliferative effects of metformin. By reducing anaplerotic flux into the TCA cycle, metformin effectively reduces the availability of mitochondrial-derived citrate for cataplerotic activity, ultimately reducing tumor cell proliferation due to substrate limitation.

Previous work has demonstrated that cancer cells are capable of adapting to mitochondrial dysfunction by engaging IDH1-dependent reductive carboxylation of α-KG rather than conventional oxidation in the TCA cycle [32,35]. This metabolic adaptation directs glutamine carbon into cytosolic acetyl-CoA pools by facilitating the production of citrate from α-KG in the cytosol, effectively bypassing the requirement for mitochondrial citrate to fuel acetyl-CoA production. Similar to previous observations [31,32], we found that metformin could stimulate reductive glutamine metabolism in tumor cells, characterized by the formation of m + 5 citrate from U-[^13^C]-glutamine. However, the overall abundance of glutamine-derived citrate generated from either oxidative (m + 4) or reductive (m + 5) pathways was reduced by <90% under conditions of metformin treatment. Thus, despite the metabolic shift towards reductive carboxylation induced by metformin treatment, this may not be a sufficient adaptation to maintain citrate production, de novo lipogenesis, and proliferation in all cell types. The rate of reductive glutamine metabolism is influenced by the citrate to α-KG ratio in cells [51]; thus, the lowering of TCA metabolite levels by metformin may affect the absolute rate of reductive carboxylation by disrupting the balance of citrate to α-KG in mitochondria.

Further evidence of a mitochondrial substrate limitation model for metformin action is supported by our data using tumor cells with dysfunctional ETC activity. 143B human osteosarcoma cells with defects in complex III activity (*cytb* mutants) are capable of cell proliferation as they actively engage mitochondrial-independent pathways of biosynthesis, including reductive glutamine metabolism for lipid biosynthesis [32]. 143B*cytb* cells generate the majority of their de novo synthesized palmitate from glutamine, a process that is insensitive to metformin treatment. When metformin is administered to these cells, there is no effective “target” for the drug, as they have altered their pathway of citrate biosynthesis to bypass the TCA cycle entirely. In contrast, cells with functional mitochondria primarily use glucose-derived citrate for palmitate biosynthesis and are unable to engage extramitochondrial citrate biosynthesis at levels needed to maintain lipid production, contributing to metformin sensitivity. These metabolic differences directly impact tumor cell proliferation: while 143B*wt* cells display a dramatic drop in glucose- and glutamine-dependent citrate and lipid production in response to metformin treatment, 143B*cytb* cells were largely unaffected, even at metformin doses that promote greater than 80% suppression of proliferation in control cells. The proliferation of both 143B*wt* and 143B*cytb* cells was sensitive to ACL inhibition, arguing that the generation of cytosolic acetyl-CoA is key for maintaining tumor cell proliferation regardless of the source of citrate.

One challenge when considering the use of biguanides for cancer treatment is identifying genetic or metabolic properties in tumors that confer sensitivity or resistance to biguanide treatment. Phenformin can confer a cytotoxic response in tumors defective in LKB1-AMPK signaling [46], and can synergize with targeted therapies to enhance therapeutic response in specific tumor types [52]. Recent work suggests that tumor cells with reduced ability to engage OXPHOS under metabolic stress, due to impaired glucose utilization or mitochondrial DNA (mtDNA) mutations in complex I genes, are also sensitive to the energy-depleting effects of biguanides [39]. One implication of our data is that metabolic adaptations that reduce the reliance of tumor cells on mitochondria for anabolic growth can contribute to resistance to biguanide treatment. One impact of these findings is that metabolic adaptations to hypoxia may contribute to biguanide resistance. Hypoxia restricts glucose and glutamine entry into the TCA cycle through HIF-1α-dependent effects on pyruvate dehydrogenase (PDH) and α-KG dehydrogenase (α-KGDH) activity, respectively [53–55]. To compensate, tumor cells use reductive glutamine metabolism to maintain acetyl-CoA production and lipid biosynthesis when growing under hypoxic conditions [34,35], effectively adopting the metabolic profile of cancer cells with mitochondrial dysfunction. 143B*wt* cells, which normally arrest cell proliferation in response to metformin, become resistant to the antiproliferative effects of metformin when cultured under hypoxia. These results may explain the limited effectiveness of metformin as a single agent or in combination with chemotherapeutic drugs under hypoxic conditions [56]. Our results suggest that targeting ACL activity may be an effective strategy to overcome biguanide resistance.

Together, our data argue against current models of metformin action through the activation of metabolic checkpoints. Rather, our data indicate that the cytostatic effects of metformin are due to restriction of important anaplerotic substrates required for TCA cycle-dependent biosynthesis. These observations provide both new insight into the mechanism of metformin action on tumor cells but also highlight metabolic adaptations engaged by tumor cells to bypass the antiproliferative effects of biguanides.

## Materials and Methods

### Cell Lines and Cell Culture

H1299 and HCT116 tumor cells were obtained from the ATCC (Manassas, VA, USA). A549 [46] and 143B osteosarcoma cells [32] have been previously described. Primary MEFs deficient for AMPKα1 and α2 or LKB1 were generated by timed mating and immortalized with SV40 Large T Antigen as previously described [57]. MEFs deficient for 4EBP1 and 4EBP2 were kindly provided by Masahiro Morita and Nahum Sonenberg [30,44]. Cells were cultured in complete growth medium (DMEM (A549 cells) or RPMI (H1299 cells)) supplemented with 10% fetal bovine serum (FBS), 20,000 U/ml penicillin, 7 mM streptomycin, 200 mM glutamine, and nonessential amino acids (for H1299 cells). Cells were grown at 37°C in a humidified atmosphere supplemented with 5% (v/v) CO_2_. Metformin and phenformin were prepared fresh and added to cell cultures at the indicated concentrations for up to 72 h.

### Cell Growth and Cell Death Assays

Cells were either seeded in 96- (8,000 cells/well) or 384-well plates (500 cells/well) in growth medium. After 24 h, medium was replaced with fresh growth medium containing various doses of either metformin or phenformin as indicated. For hypoxia treatments cells were cultured for up to 72 h under 1% O_2_ tension in a Heracell Tri-gas incubator (Forma Scientific). Cells were fixed with 4% paraformaldehyde at 24, 48, and 72 h for 20 min at room temperature, followed by staining with crystal violet (0.05% (w/v) crystal violet and 20% (v/v) 95% ethanol) for 30 min, solubilization in PBS containing 1% SDS for 1 h, and then analyzed at 595 nm on a Spectramax plate reader (Molecular Devices, Sunnyvale, CA, USA). Alternatively, cells were seeded in 384 well plates (500 cells/well) in growth medium. After 24 h, growth medium was replaced with fresh medium containing various doses of metformin. Cells were fixed with 4% formaldehyde, stained with Hoechst DNA stain, and cell number was determined by nuclei counting. Images were taken using an Operetta High Content Imaging System and analyzed using Harmony High Content Imaging and Analysis Software, both from Perkin Elmer (Waltham, MA, USA). Cell viability was assessed by viability dye exclusion using flow cytometry as previously described [24]. Viability was determined relative to cells subjected to control treatment 48–72 h following biguanide treatment. Viability was assessed using flow cytometry and measuring propidium iodide incorporation. Transient knockdown of ACL was achieved using SMARTpool ON-TARGETplus siRNA (GE Dharmacon, Layette, CO, USA). Briefly, 50 nM siRNA was incubated for 20 min with RNAimax in OptiMEM medium (Life Technologies) in a 12-well plate, followed by seeding of cells (60,000 cells/well). Cells were transfected a second time 48 h later and plated for assays the following day.

### Immunoblotting

Cells were lysed in modified CHAPS buffer (10mM Tris·HCl, 1 mM MgCl2, 1 mM EGTA, 0.5 mM CHAPS, 10% glycerol, 5 mM NaF) supplemented with the following protease additives: protease and phosphatase tablets (Roche), DTT (1 μg/mL), and benzamidine (1 μg/mL). Cleared lysates were resolved by SDS/PAGE, transferred to nitrocellulose, and incubated with primary antibodies. Primary antibodies to AMPKα (pT172-specific and total), S6 ribosomal protein (pS235/236-specific and total), 4E-BP1 (pT37/46-specific and total), GAPDH, as well as HRP-conjugated anti-rabbit secondary antibodies were obtained from Cell Signaling Technology (Danvers, MA, USA).

### Seahorse XF96 Respirometry and Metabolic Assays

OCR and ECAR of cells were measured using an XF96 Extracellular Flux Analyzer (Seahorse Bioscience) using established protocols [24,58]. In brief, cells were plated at 1 × 10^4^ per well in 140 μL of nonbuffered DMEM containing 25 mM glucose and 2 mM glutamine. Cells were incubated in a CO_2_-free incubator at 37°C for 2 h to allow for temperature and pH equilibration before loading into the XF96 apparatus. XF assays consisted of sequential mix (3 min), pause (3 min), and measurement (5 min) cycles, allowing for determination of OCR/ECAR every 10 min. H1299 cells were pre-treated with 5 mM metformin for 6 h. Primary MEFs deficient for AMPKα were pretreated with 10 mM metformin for 24 h. Glucose and lactate levels in culture medium were measured using a Flux Bioanalyzer (NOVA Biomedical) as previously described [24].

### GC-MS Analysis of ^13^C Metabolites

Cellular metabolites were extracted and analyzed by gas chromatography-mass spectrometry (GC-MS) using previously described protocols [26,58–60]. For mass isotopomer analysis, cells were incubated in glucose and glutamine-free DMEM containing 10% dialyzed FBS and uniformly labeled [^13^C]-glucose or [^13^C]-glutamine (Cambridge Isotope Laboratories). Extraction of metabolites for GC-MS analysis was conducted as outlined previously [26,58]. For TCA cycle analysis, cells (2–5 × 10^6^ per 10-cm dish) were cultured in control medium or medium containing 5 mM metformin for 6 h, followed by a 2 h incubation with the tracer. Lysis in ice-cold 80% methanol and sonication were then performed following the pulse. Before the samples were dried down, D-myristic acid (750 ng/sample) was added as an internal standard. Dried samples were then dissolved in 30 μL methoxyamine hydrochloride (10 mg/ml) in pyridine and derivatized as tert-butyldimethylsilyl (TBDMS) esters using 70 μL N-(*tert*-butyldimethylsilyl)-N-methyltrifluoroacetamide (MTBSTFA).

For fatty acid profiles, cells were treated with 5 mM metformin (H1299 cells) or 10 mM metformin (143B cells) for 48 h followed by culture with U-[^13^C]-glucose or U-[^13^C]-glutamine for an additional 24 h. Triglycerides and other lipids were extracted using a modified Folch method [61] substituting methylene chloride for chloroform. Following extraction, the organic layer was isolated, dried in a warm N_2_ stream, and saponified in potassium hydroxide overnight at 60°C. The fatty acids were re-extracted and dried. Dried samples were then dissolved in 30 μL pyridine and derivatized TBDMS esters using 70 μL MTBSTFA.

For both TCA and fatty acids analysis, an Agilent 5975C GC-MS equipped with a DB-5MS + DG (30 m x 250 μm x 0.25 μm) capillary column (Agilent J&W, Santa Clara, CA, USA) was used. All data were collected by electron impact set at 70 eV. A total of 1 μL of the derivatized sample was injected in the GC in splitless mode with inlet temperature set to 280°C, using helium as a carrier gas with a flow rate of 1.5512 mL/min (rate at which myristic acid elutes at 17.94 min). The quadrupole was set at 150°C and the GC/MS interface at 285°C. The oven program for all metabolite analyses started at 60°C held for 1 min, then increasing at a rate of 10°C/min until 320°C. Bake-out was at 320°C for 10 min. Sample data were acquired both in scan (1–600 m/z) and selected ion monitoring (SIM) modes [59].

Mass isotopomer distribution for TCA cycle and fatty acid intermediates was determined using a custom algorithm developed at McGill University [59]. Briefly, the atomic composition of the TBDMS-derivatized metabolite fragments (M-57) was determined, and, using the algorithm, matrices correcting for natural contribution of isotopomer enrichment were generated for each metabolite. After correction for natural abundance, a comparison was made between nonlabeled metabolite abundances (^12^C) and metabolite abundances which were synthesized from the tracer which was pulsed into the cells (^13^C). Metabolite abundance was expressed relative to the internal standard (D-myristic acid) and normalized to cell number. For palmitate analysis of labeling patterns, ISA technique was performed.

### Metabolite Profiling by LC-MS

Cellular metabolites were extracted and analyzed LC-MS using previously described protocols [58–60]. Liquid chromatography was performed using a 1290 Infinity ultraperformance LC system (Agilent Technologies, Santa Clara, CA, USA) equipped with a Scherzo 3 μm, 3.0 × 150mm SM-C18 column (Imtakt Corp, Japan). The column temperature was maintained at 10°C, and the mobile phases A and B consisted of water containing 5 and 200 mM ammonium acetate, respectively. The chromatographic gradient started at 100% mobile phase (A) with a 5 min gradient to 100% (B). This was followed by a 5 min hold time at 100% mobile phase B at a flow rate of 0.4ml/min. A subsequent re-equilibration time (6 min) was performed before the next injection. Sample volumes of 5 μl were injected for LC-MS analysis. LC-MS analysis was performed on an Agilent 6540 UHD Accurate-Mass Q-TOF mass spectrometer (Agilent Technologies, Santa Clara, CA, USA). Analyte ionization was accomplished using an electrospray ionization source (ESI) in both positive and negative polarities. The source operating conditions were set at 325°C and 9l/min for gas temperature and flow respectively, nebulizer pressure was set at 40 psi and capillary voltage was set a 4.0 kV. Reference masses 121.0509, 922.0099, 1033.9881 were introduced into the source through a secondary spray nozzle to ensure accurate mass. MS data were acquired in full scan mode mass range: m/z 100–1,000; scan time: 1.4s; data collection: centroid and profile. Retention times, and accurate mass for each compound were confirmed against authentic standards as well as matched unlabeled cell extracts grown under the same conditions when cells were undergoing SITA. Data were quantified by integrating the area underneath the curve of each compound using MassHunter Qual (Agilent Technologies, Santa Clara, CA, USA). Each metabolite’s accurate mass ion and subsequent isotopic ions were extracted (EIC) using a 10 ppm window.

### Isotopomer Spectral Analysis (ISA) of Palmitate Mass Isotopomer Distributions

Isotopomer spectral analysis (ISA) was used to quantify the contribution of ^13^C-glucose, ^13^C-glutamine, and ^13^C-acetate to lipogenic acetyl-CoA pools [62]. ISA extracts this contribution from the experimentally measured palmitate MIDs by applying a system of equations describing the mixing of labeled and unlabeled acetyl-CoA, de novo palmitate synthesis, and the mixture of the newly synthesized palmitate with pre-existing, unlabeled palmitate. In the current ISA model, palmitate synthesis is modeled as the polymerization of eight two-carbon acetyl-CoA units. ISA was performed using the INCA software package [63], which minimizes the sum of squares residuals between the experimentally derived and simulated palmitate MIDs. 95% confidence intervals were calculated for the best fit solution [64].

### Statistical Analysis

Data are presented as mean ± SD for technical replicates, or mean ± SEM for biological replicates, and analyzed using unpaired Student’s *t* test. Statistical significance is indicated in all figures by the following annotations: *, *p* < 0.05; **, *p* < 0.01; ***, *p* < 0.001; *p* < 0.0001.

## Supporting Information

S1 Data Excel file containing the raw data for Fig 1. (XLSX)

S2 Data Excel file containing the raw data for Fig 2. (XLSX)

S3 Data Excel file containing the raw data for Fig 3. (XLSX)

S4 Data Excel file containing the raw data for Fig 4. (XLSX)

S5 Data Excel file containing the raw data for Fig 5. (XLSX)

S6 Data Excel file containing the raw data for Fig 6. (XLSX)

S7 Data Excel file containing the raw data for Fig 7. (XLSX)

S8 Data Excel file containing the raw data for S1 Fig. (XLSX)

S9 Data Excel file containing the raw data for S2 Fig. (XLSX)

S10 Data Excel file containing the raw data for S3 Fig. (XLSX)

S11 Data Excel file containing the raw data for S4 Fig. (XLSX)

S12 Data Excel file containing the raw data for S5 Fig. (XLSX)

S13 Data Excel file containing the raw data for S6 Fig. (XLSX)

S1 Fig Metformin inhibits cancer cell proliferation and alters glucose metabolism and oxygen consumption. Related to Fig 1. **A.** H1299 cell viability measured using propidium iodide incorporation. Cells were treated with (+) or without (−) 5 mM metformin for 72 h in regular growth media. Data normalized to control conditions. Data presented as mean ± SD for triplicate samples and are representative of three independent experiments. **B**. Proliferation of HCT116 cells treated with varying doses of metformin for 72 h. Cell numbers are expressed relative to cell counts in control conditions (0 mM metformin). Each data point represents the mean ± SEM for triplicate samples. **C.** Nucleotide abundance of H1299 cells after 14 h of treatment with varying doses of metformin. Abundances were measured by LC-MS. **D–E**. OCR (**D**) and ECAR (**E**) of H1299 cells cultured for 6 h in the presence or absence of 5 mM metformin. The data represent the mean ± SEM for each condition (*n* = 6 samples per condition) and are representative of three independent experiments. **F–G**. Glucose consumption (**F**) and lactate production (**G**) of H1299 cells after 48 h of treatment with or without metformin (5 mM). The data represent the mean ± SEM for each condition (*n* = 3 samples per condition) and are representative of three independent experiments. *, *p* < 0.05; **, *p* < 0.01; ***, *p* < 0.001; *p* < 0.0001. Raw data for this figure can be found in S8 Data.
(TIF)

S2 Fig Phenformin inhibits cellular proliferation independent of LKB1 and 4EBP1/2. Related to Fig 2. **A–B.** Proliferation of A549 cells expressing empty vector (A549/Vec) or LKB1 vector (A549/LKB1) (**A**) and MEFs expressing WT 4EBP1/2 (4EBP1/2 WT) or knockout for 4EBP1/2 (4EBP1/2 double knockout [DKO]) (**B**), treated with varying doses of phenformin for 72 h. Cell numbers are expressed relative to cell counts in control conditions (0 μM phenformin). The data represent the mean ± SEM for each condition (*n* = 12 samples per condition), and are representative of two independent experiments. Raw data for this figure can be found in S9 Data.
(TIF)

S3 Fig Metformin inhibits glucose and glutamine incorporation into TCA cycle metabolites. Related to Fig 3. **A–B.** H1299 cells were treated with (+) or without (−) 5 mM metformin for 6 h, followed by culture with U-[^13^C]-glucose (**A**) or U-[^13^C]-glutamine (**B**) for an additional 2 h. Shown is the relative abundance (left panel) and isotopomer distribution (right panel) for succinate (top panels) and fumarate (bottom panel) under each culture condition. Cells were then extracted and analyzed by GC-MS. **C.** Relative citrate abundance of MEFs, WT or KO for AMPK, after treatment with (+) or without (−) 5 mM metformin for 6 h, followed by a culture with U-[^13^C]-glucose or U-[^13^C]-glutamine for an additional 2 h. Data represents mean ± SEM for each condition (*n* = 3). Data shown is representative of three independent experiments. *, *p* < 0.05; **, p<0.01; ***, p<0.001; p<0.0001. Raw data for this figure can be found in S10 Data.
(TIF)

S4 Fig Metformin treatment results in reduced fatty acid abundance. Related to Fig 4. **A.** Relative abundance of palmitate in H1299 cells following culture either in the presence or absence of metformin (5 mM) for 72 h. Cells were extracted following the exposure and analyzed using GC-MS. Data represent mean ± SEM for each condition (*n* = 3). Data shown is representative of three independent experiments. *, *p* < 0.05 **B–C.** Relative lipogenic acetyl-CoA and palmitate mass isotopomer distribution derived from U-[^13^C]-glucose **(B)** or U-[^13^C]-glutamine **(C)** in MEFs both WT (white) and KO (red) for AMPK after treatment for 48 h with (hashed bars) or without (open bars) 5 mM metformin and pulsed of 24 h. **D.** Relative lipogenic acetyl-CoA and palmitate abundance derived from U-[^13^C]-glucose in H1299 cells after treatment with (black bars) or without (white bars) 5 mM metformin for 48 h followed by glucose pulse with (+) or without (−) 200 uM etomoxir for 24 h. Data represent mean ± SD for each condition (*n* = 3). Data shown is representative of two independent experiments. Raw data for this figure can be found in S11 Data.
(TIF)

S5 Fig Cancer cells undergoing mitochondrial-independent macromolecular biosynthesis are resistant to metformin treatment. Related to Fig 5. **A–B.** OCR (**A**) and ECAR (**B**) of 143B osteosarcoma cells cultured with (+) or without (–) metformin (5 mM) for 6 h. **C**. Relative metabolite abundance of 143B*wt* (white bars) and 143B*cytb* (black bars) cells cultured with or without metformin (10 mM) for 12 h. Data expressed as a ratio of metformin treated cells over control conditions (0 mM metformin). Data represents mean ± SEM for each condition (*n* = 3). Data is representative of two independent experiments. **D**. Isotopomer distribution of U-[^13^C]-glucose- and U-[^13^C]-glutamine-derived palmitate of 143B*wt* and 143B*cytb* cells cultured with (black bars) or without (white bars) metformin (10mM) treatment. Cells were cultured either with or without 10mM metformin for 48 h followed by a 24 h pulse with either U-[^13^C]-glucose (top panel) or U-[^13^C]-glutamine (bottom panel). Data represents mean ± SEM for each condition (*n* = 3). Data shown is representative of three independent experiments. *, *p* < 0.05; ***, *p* < 0.001; *p* < 0.0001. Raw data for this figure can be found in S12 Data.
(TIF)

S6 Fig Cancer cells require ACL for continued proliferation in control conditions. Related to Fig 6. **A.** 143B cell viability measured using propidium iodide incorporation. Cells were treated with (black bars) or without (white bars) 5 mM metformin for 72 h in regular growth media. Data normalized to control conditions. Data presented as mean ± SD for triplicate samples and are representative of three independent experiments. **B.** Proliferation of 143B*wt* and 143B*cytb* cells with control siRNA (CTL) or ACL siRNA (*ACL*). The data represent the mean ± SEM for each condition (*n* = 12 samples per condition), and are representative of two independent experiments. Cell numbers normalized to siRNA CTL conditions. **C.** Relative abundance of U-[^13^C]-glutamine-derived citrate in 143B*wt* cells with (+) or without (−) 5 mM metformin with control siRNA (-) or ACL siRNA (+) treatment. Cells were treated with 5 mM metformin for 6 h followed by a glutamine pulse for 6 h. Data represents mean ± SEM for each condition (*n* = 3). Raw data for this figure can be found in S13 Data.
(TIF)

## Abbreviations

143B*wt*: 143B human osteosarcoma cells with intact complex III activity
143B*cytb*: 143B human osteosarcoma cells with defective complex III activity
αKG: α-ketoglutarate
α-KGDH: α-ketoglutarate dehydrogenase
ACL: ATP citrate lyase
AMPK: AMP-activated protein kinase
DKO: double knockout
ECAR: extracellular acidification rate
ETC: electron transport chain
FA: fatty acid
GC-MS: gas chromatography-mass spectrometry
IDH: isocitrate dehydrogenase
IGF-1: insulin-like growth factor-1
KO: knockout
MEFs: mouse embryonic fibroblasts
mtDNA: mitochondrial DNA
mTOR: mammalian target of rapamycin
NSCLC: non-small cell lung cancer
OCR: O_2_ consumption rate
OXPHOS: oxidative phosphorylation
PDH: pyruvate dehydrogenase
SITA: stable isotope tracer analysis
TCA: Tricarboxylic Acid
WT: wild type

## References

[1] Andrzejewski S, Gravel SP, Pollak M, St-Pierre J. Metformin directly acts on mitochondria to alter cellular bioenergetics. Cancer & metabolism. 2014;2:12. 25184038 10.1186/2049-3002-2-12 PMC4147388

[2] Bridges HR, Jones AJ, Pollak MN, Hirst J. Effects of metformin and other biguanides on oxidative phosphorylation in mitochondria. Biochem J. 2014;462(3):475–87. 10.1042/BJ20140620 25017630 PMC4148174

[3] Owen MR, Doran E, Halestrap AP. Evidence that metformin exerts its anti-diabetic effects through inhibition of complex 1 of the mitochondrial respiratory chain. Biochem J. 2000;348 Pt 3:607–14. Epub 2000/06/07. 10839993 PMC1221104

[4] El-Mir MY, Nogueira V, Fontaine E, Averet N, Rigoulet M, Leverve X. Dimethylbiguanide inhibits cell respiration via an indirect effect targeted on the respiratory chain complex I. J Biol Chem. 2000;275(1):223–8. Epub 2000/01/05. 10617608 10.1074/jbc.275.1.223

[5] Inzucchi SE, Maggs DG, Spollett GR, Page SL, Rife FS, Walton V, et al Efficacy and metabolic effects of metformin and troglitazone in type II diabetes mellitus. N Engl J Med. 1998;338(13):867–72. Epub 1998/03/27. 9516221 10.1056/NEJM199803263381303

[6] Stumvoll M, Nurjhan N, Perriello G, Dailey G, Gerich JE. Metabolic effects of metformin in non-insulin-dependent diabetes mellitus. N Engl J Med. 1995;333(9):550–4. Epub 1995/08/31. 7623903 10.1056/NEJM199508313330903

[7] Perriello G, Misericordia P, Volpi E, Santucci A, Santucci C, Ferrannini E, et al Acute antihyperglycemic mechanisms of metformin in NIDDM. Evidence for suppression of lipid oxidation and hepatic glucose production. Diabetes. 1994;43(7):920–8. Epub 1994/07/01. 8013758 10.2337/diab.43.7.920

[8] Shaw RJ, Lamia KA, Vasquez D, Koo SH, Bardeesy N, Depinho RA, et al The kinase LKB1 mediates glucose homeostasis in liver and therapeutic effects of metformin. Science. 2005;310(5754):1642–6. Epub 2005/11/26. 16308421 10.1126/science.1120781 PMC3074427

[9] Evans JM, Donnelly LA, Emslie-Smith AM, Alessi DR, Morris AD. Metformin and reduced risk of cancer in diabetic patients. Bmj. 2005;330(7503):1304–5. Epub 2005/04/26. 15849206 10.1136/bmj.38415.708634.F7 PMC558205

[10] Quinn BJ, Kitagawa H, Memmott RM, Gills JJ, Dennis PA. Repositioning metformin for cancer prevention and treatment. Trends in endocrinology and metabolism: TEM. 2013;24(9):469–80. Epub 2013/06/19. 10.1016/j.tem.2013.05.004 23773243

[11] Pollak MN. Investigating metformin for cancer prevention and treatment: the end of the beginning. Cancer discovery. 2012;2(9):778–90. 10.1158/2159-8290.CD-12-0263 22926251

[12] Wheaton WW, Weinberg SE, Hamanaka RB, Soberanes S, Sullivan LB, Anso E, et al Metformin inhibits mitochondrial complex I of cancer cells to reduce tumorigenesis. eLife. 2014;3:e02242 10.7554/eLife.02242 24843020 PMC4017650

[13] Shackelford DB, Shaw RJ. The LKB1-AMPK pathway: metabolism and growth control in tumour suppression. Nat Rev Cancer. 2009;9(8):563–75. Epub 2009/07/25. 10.1038/nrc2676 19629071 PMC2756045

[14] Zhou G, Myers R, Li Y, Chen Y, Shen X, Fenyk-Melody J, et al Role of AMP-activated protein kinase in mechanism of metformin action. J Clin Invest. 2001;108(8):1167–74. 11602624 10.1172/JCI13505 PMC209533

[15] Fryer LG, Parbu-Patel A, Carling D. The Anti-diabetic drugs rosiglitazone and metformin stimulate AMP-activated protein kinase through distinct signaling pathways. J Biol Chem. 2002;277(28):25226–32. Epub 2002/05/08. 11994296 10.1074/jbc.M202489200

[16] Hawley SA, Gadalla AE, Olsen GS, Hardie DG. The antidiabetic drug metformin activates the AMP-activated protein kinase cascade via an adenine nucleotide-independent mechanism. Diabetes. 2002;51(8):2420–5. Epub 2002/07/30. 12145153 10.2337/diabetes.51.8.2420

[17] Buzzai M, Jones RG, Amaravadi RK, Lum JJ, DeBerardinis RJ, Zhao F, et al Systemic treatment with the antidiabetic drug metformin selectively impairs p53-deficient tumor cell growth. Cancer Res. 2007;67(14):6745–52. Epub 2007/07/20. 17638885 10.1158/0008-5472.CAN-06-4447

[18] Huang X, Wullschleger S, Shpiro N, McGuire VA, Sakamoto K, Woods YL, et al Important role of the LKB1-AMPK pathway in suppressing tumorigenesis in PTEN-deficient mice. Biochem J. 2008;412(2):211–21. 10.1042/BJ20080557 18387000

[19] Kalender A, Selvaraj A, Kim SY, Gulati P, Brule S, Viollet B, et al Metformin, independent of AMPK, inhibits mTORC1 in a rag GTPase-dependent manner. Cell Metab. 2010;11(5):390–401. 10.1016/j.cmet.2010.03.014 20444419 PMC3081779

[20] Dowling RJ, Zakikhani M, Fantus IG, Pollak M, Sonenberg N. Metformin inhibits mammalian target of rapamycin-dependent translation initiation in breast cancer cells. Cancer Res. 2007;67(22):10804–12. Epub 2007/11/17. 18006825 10.1158/0008-5472.CAN-07-2310

[21] Liu X, Chhipa RR, Pooya S, Wortman M, Yachyshin S, Chow LM, et al Discrete mechanisms of mTOR and cell cycle regulation by AMPK agonists independent of AMPK. Proc Natl Acad Sci U S A. 2014;111(4):E435–44. Epub 2014/01/30. 10.1073/pnas.1311121111 24474794 PMC3910576

[22] Zakikhani M, Dowling R, Fantus IG, Sonenberg N, Pollak M. Metformin is an AMP kinase-dependent growth inhibitor for breast cancer cells. Cancer Res. 2006;66(21):10269–73. 17062558 10.1158/0008-5472.CAN-06-1500

[23] Vazquez-Martin A, Oliveras-Ferraros C, Menendez JA. The antidiabetic drug metformin suppresses HER2 (erbB-2) oncoprotein overexpression via inhibition of the mTOR effector p70S6K1 in human breast carcinoma cells. Cell Cycle. 2009;8(1):88–96. 19106626 10.4161/cc.8.1.7499

[24] Vincent EE, Coelho PP, Blagih J, Griss T, Viollet B, Jones RG. Differential effects of AMPK agonists on cell growth and metabolism. Oncogene. 2014. Epub 2014/09/23. 10.1038/onc.2014.301 PMC4980123 25241895

[25] Wu M, Neilson A, Swift AL, Moran R, Tamagnine J, Parslow D, et al Multiparameter metabolic analysis reveals a close link between attenuated mitochondrial bioenergetic function and enhanced glycolysis dependency in human tumor cells. Am J Physiol Cell Physiol. 2007;292(1):C125–36. Epub 2006/09/15. 16971499 10.1152/ajpcell.00247.2006

[26] Faubert B, Boily G, Izreig S, Griss T, Samborska B, Dong Z, et al AMPK is a negative regulator of the Warburg effect and suppresses tumor growth in vivo. Cell Metab. 2013;17(1):113–24. 10.1016/j.cmet.2012.12.001 23274086 PMC3545102

[27] Laderoute KR, Amin K, Calaoagan JM, Knapp M, Le T, Orduna J, et al 5'-AMP-activated protein kinase (AMPK) is induced by low-oxygen and glucose deprivation conditions found in solid-tumor microenvironments. Mol Cell Biol. 2006;26(14):5336–47. Epub 2006/07/01. 16809770 10.1128/MCB.00166-06 PMC1592699

[28] Shaw RJ, Kosmatka M, Bardeesy N, Hurley RL, Witters LA, DePinho RA, et al The tumor suppressor LKB1 kinase directly activates AMP-activated kinase and regulates apoptosis in response to energy stress. Proc Natl Acad Sci U S A. 2004;101(10):3329–35. 14985505 10.1073/pnas.0308061100 PMC373461

[29] Tzatsos A, Kandror KV. Nutrients suppress phosphatidylinositol 3-kinase/Akt signaling via raptor-dependent mTOR-mediated insulin receptor substrate 1 phosphorylation. Mol Cell Biol. 2006;26(1):63–76. Epub 2005/12/16. 16354680 10.1128/MCB.26.1.63-76.2006 PMC1317643

[30] Dowling RJ, Topisirovic I, Alain T, Bidinosti M, Fonseca BD, Petroulakis E, et al mTORC1-mediated cell proliferation, but not cell growth, controlled by the 4E-BPs. Science. 2010;328(5982):1172–6. Epub 2010/05/29. 10.1126/science.1187532 20508131 PMC2893390

[31] Fendt SM, Bell EL, Keibler MA, Davidson SM, Wirth GJ, Fiske B, et al Metformin decreases glucose oxidation and increases the dependency of prostate cancer cells on reductive glutamine metabolism. Cancer Res. 2013;73(14):4429–38. Epub 2013/05/21. 10.1158/0008-5472.CAN-13-0080 23687346 PMC3930683

[32] Mullen AR, Wheaton WW, Jin ES, Chen PH, Sullivan LB, Cheng T, et al Reductive carboxylation supports growth in tumour cells with defective mitochondria. Nature. 2012;481(7381):385–8. 10.1038/nature10642 PMC3262117 22101431

[33] Hatzivassiliou G, Zhao F, Bauer DE, Andreadis C, Shaw AN, Dhanak D, et al ATP citrate lyase inhibition can suppress tumor cell growth. Cancer Cell. 2005;8(4):311–21. 16226706 10.1016/j.ccr.2005.09.008

[34] Wise DR, Ward PS, Shay JE, Cross JR, Gruber JJ, Sachdeva UM, et al Hypoxia promotes isocitrate dehydrogenase-dependent carboxylation of alpha-ketoglutarate to citrate to support cell growth and viability. Proc Natl Acad Sci U S A. 2011;108(49):19611–6. Epub 2011/11/23. 10.1073/pnas.1117773108 22106302 PMC3241793

[35] Metallo CM, Gameiro PA, Bell EL, Mattaini KR, Yang J, Hiller K, et al Reductive glutamine metabolism by IDH1 mediates lipogenesis under hypoxia. Nature. 2012;481(7381):380–4. Epub 2011/11/22. 10.1038/nature10602 PMC3710581 22101433

[36] Weinberg F, Hamanaka R, Wheaton WW, Weinberg S, Joseph J, Lopez M, et al Mitochondrial metabolism and ROS generation are essential for Kras-mediated tumorigenicity. Proc Natl Acad Sci U S A. 2010;107(19):8788–93. 10.1073/pnas.1003428107 20421486 PMC2889315

[37] Rana M, de Coo I, Diaz F, Smeets H, Moraes CT. An out-of-frame cytochrome b gene deletion from a patient with parkinsonism is associated with impaired complex III assembly and an increase in free radical production. Ann Neurol. 2000;48(5):774–81. 11079541

[38] Kamphorst JJ, Chung MK, Fan J, Rabinowitz JD. Quantitative analysis of acetyl-CoA production in hypoxic cancer cells reveals substantial contribution from acetate. Cancer & metabolism. 2014;2:23. Epub 2015/02/12. 25671109 10.1186/2049-3002-2-23 PMC4322440

[39] Birsoy K, Possemato R, Lorbeer FK, Bayraktar EC, Thiru P, Yucel B, et al Metabolic determinants of cancer cell sensitivity to glucose limitation and biguanides. Nature. 2014;508(7494):108–12. 10.1038/nature13110 24670634 PMC4012432

[40] Lunt SY, Vander Heiden MG. Aerobic glycolysis: meeting the metabolic requirements of cell proliferation. Annual review of cell and developmental biology. 2011;27:441–64. Epub 2011/10/12. 10.1146/annurev-cellbio-092910-154237 21985671

[41] Deberardinis RJ, Sayed N, Ditsworth D, Thompson CB. Brick by brick: metabolism and tumor cell growth. Curr Opin Genet Dev. 2008;18(1):54–61. Epub 2008/04/05. 10.1016/j.gde.2008.02.003 18387799 PMC2476215

[42] Ahn CS, Metallo CM. Mitochondria as biosynthetic factories for cancer proliferation. Cancer & metabolism. 2015;3(1):1. 25621173 10.1186/s40170-015-0128-2 PMC4305394

[43] Menendez JA, Oliveras-Ferraros C, Cufi S, Corominas-Faja B, Joven J, Martin-Castillo B, et al Metformin is synthetically lethal with glucose withdrawal in cancer cells. Cell Cycle. 2012;11(15):2782–92. 10.4161/cc.20948 22809961

[44] Morita M, Gravel SP, Chenard V, Sikstrom K, Zheng L, Alain T, et al mTORC1 controls mitochondrial activity and biogenesis through 4E-BP-dependent translational regulation. Cell Metab. 2013;18(5):698–711. Epub 2013/11/12. 10.1016/j.cmet.2013.10.001 24206664

[45] Duvel K, Yecies JL, Menon S, Raman P, Lipovsky AI, Souza AL, et al Activation of a metabolic gene regulatory network downstream of mTOR complex 1. Mol Cell. 2010;39(2):171–83. Epub 2010/07/31. 10.1016/j.molcel.2010.06.022 20670887 PMC2946786

[46] Shackelford DB, Abt E, Gerken L, Vasquez DS, Seki A, Leblanc M, et al LKB1 Inactivation Dictates Therapeutic Response of Non-Small Cell Lung Cancer to the Metabolism Drug Phenformin. Cancer Cell. 2013;23(2):143–58. Epub 2013/01/29. 10.1016/j.ccr.2012.12.008 23352126 PMC3579627

[47] Bungard D, Fuerth BJ, Zeng PY, Faubert B, Maas NL, Viollet B, et al Signaling kinase AMPK activates stress-promoted transcription via histone H2B phosphorylation. Science. 2010;329(5996):1201–5. Epub 2010/07/22. 10.1126/science.1191241 20647423 PMC3922052

[48] Liu L, Ulbrich J, Muller J, Wustefeld T, Aeberhard L, Kress TR, et al Deregulated MYC expression induces dependence upon AMPK-related kinase 5. Nature. 2012;483(7391):608–12. Epub 2012/03/31. 10.1038/nature10927 22460906

[49] DeBerardinis RJ, Lum JJ, Hatzivassiliou G, Thompson CB. The biology of cancer: metabolic reprogramming fuels cell growth and proliferation. Cell Metab. 2008;7(1):11–20. Epub 2008/01/08. 10.1016/j.cmet.2007.10.002 18177721

[50] Bauer DE, Hatzivassiliou G, Zhao F, Andreadis C, Thompson CB. ATP citrate lyase is an important component of cell growth and transformation. Oncogene. 2005;24(41):6314–22. 16007201 10.1038/sj.onc.1208773

[51] Fendt SM, Bell EL, Keibler MA, Olenchock BA, Mayers JR, Wasylenko TM, et al Reductive glutamine metabolism is a function of the alpha-ketoglutarate to citrate ratio in cells. Nature communications. 2013;4:2236 Epub 2013/08/01. 10.1038/ncomms3236 23900562 PMC3934748

[52] Yuan P, Ito K, Perez-Lorenzo R, Del Guzzo C, Lee JH, Shen CH, et al Phenformin enhances the therapeutic benefit of BRAF(V600E) inhibition in melanoma. Proc Natl Acad Sci U S A. 2013;110(45):18226–31. Epub 2013/10/23. 10.1073/pnas.1317577110 24145418 PMC3831456

[53] Sun RC, Denko NC. Hypoxic regulation of glutamine metabolism through HIF1 and SIAH2 supports lipid synthesis that is necessary for tumor growth. Cell Metab. 2014;19(2):285–92. 10.1016/j.cmet.2013.11.022 24506869 PMC3920584

[54] Papandreou I, Cairns RA, Fontana L, Lim AL, Denko NC. HIF-1 mediates adaptation to hypoxia by actively downregulating mitochondrial oxygen consumption. Cell Metab. 2006;3(3):187–97. Epub 2006/03/07. 16517406 10.1016/j.cmet.2006.01.012

[55] Kim JW, Tchernyshyov I, Semenza GL, Dang CV. HIF-1-mediated expression of pyruvate dehydrogenase kinase: a metabolic switch required for cellular adaptation to hypoxia. Cell Metab. 2006;3(3):177–85. Epub 2006/03/07. 16517405 10.1016/j.cmet.2006.02.002

[56] Garofalo C, Capristo M, Manara MC, Mancarella C, Landuzzi L, Belfiore A, et al Metformin as an adjuvant drug against pediatric sarcomas: hypoxia limits therapeutic effects of the drug. PLoS One. 2013;8(12):e83832 10.1371/journal.pone.0083832 24391834 PMC3877110

[57] Jones RG, Plas DR, Kubek S, Buzzai M, Mu J, Xu Y, et al AMP-activated protein kinase induces a p53-dependent metabolic checkpoint. Mol Cell. 2005;18(3):283–93. 15866171 10.1016/j.molcel.2005.03.027

[58] Faubert B, Vincent EE, Griss T, Samborska B, Izreig S, Svensson RU, et al Loss of the tumor suppressor LKB1 promotes metabolic reprogramming of cancer cells via HIF-1alpha. Proc Natl Acad Sci U S A. 2014;111(7):2554–9. Epub 2014/02/20. 10.1073/pnas.1312570111 24550282 PMC3932920

[59] McGuirk S, Gravel SP, Deblois G, Papadopoli DJ, Faubert B, Wegner A, et al PGC-1alpha supports glutamine metabolism in breast cancer. Cancer & metabolism. 2013;1(1):22. 24304688 10.1186/2049-3002-1-22 PMC4178216

[60] Dupuy F, Griss T, Blagih J, Bridon G, Avizonis D, Ling C, et al LKB1 is a central regulator of tumor initiation and pro-growth metabolism in ErbB2-mediated breast cancer. Cancer & metabolism. 2013;1(1):18. Epub 2013/11/28. 24280377 10.1186/2049-3002-1-18 PMC4178213

[61] Folch J, Ascoli I, Lees M, Meath JA, Le BN. Preparation of lipide extracts from brain tissue. J Biol Chem. 1951;191(2):833–41. Epub 1951/08/01. 14861228

[62] Kharroubi AT, Masterson TM, Aldaghlas TA, Kennedy KA, Kelleher JK. Isotopomer spectral analysis of triglyceride fatty acid synthesis in 3T3-L1 cells. The American journal of physiology. 1992;263(4 Pt 1):E667–75. 1415685 10.1152/ajpendo.1992.263.4.E667

[63] Young JD. INCA: a computational platform for isotopically non-stationary metabolic flux analysis. Bioinformatics. 2014;30(9):1333–5. 10.1093/bioinformatics/btu015 24413674 PMC3998137

[64] Antoniewicz MR, Kelleher JK, Stephanopoulos G. Determination of confidence intervals of metabolic fluxes estimated from stable isotope measurements. Metab Eng. 2006;8(4):324–37. 16631402 10.1016/j.ymben.2006.01.004

