# Supplementary figures and images for: Metformin Antagonizes Cancer Cell Proliferation by Suppressing Mitochondrial-Dependent Biosynthesis
Source: PLoS Biol. 2015 Dec 1;13(12):e1002309. doi: 10.1371/journal.pbio.1002309 (PMC4666657; doi:10.1371/journal.pbio.1002309)

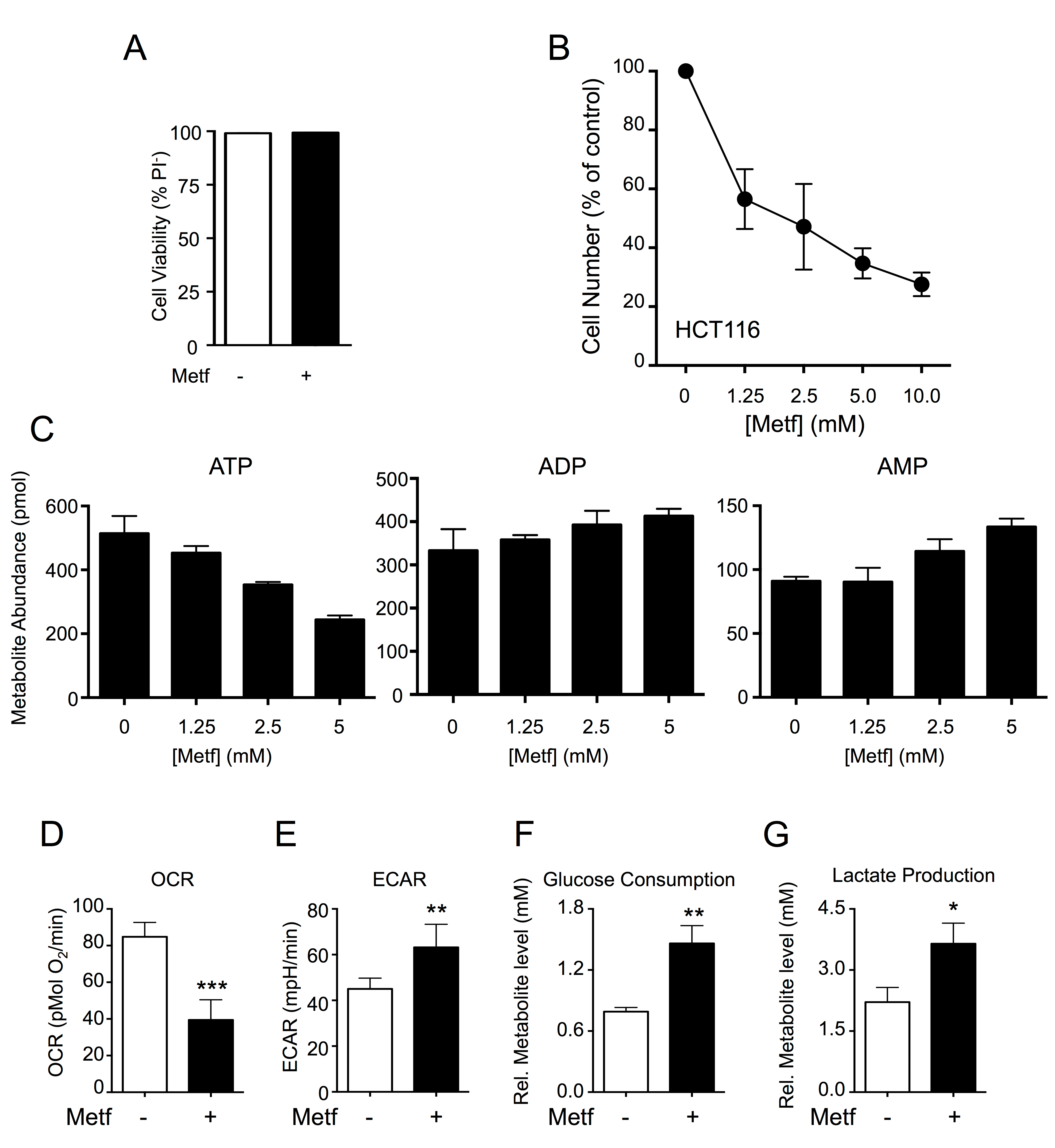

Supplement: S1 Fig — Related to Fig 1. A. H1299 cell viability measured using propidium iodide incorporation. Cells were treated with (+) or without (−) 5 mM metformin for 72 h in regular growth media. Data normalized to control conditions. Data presented as mean ± SD for triplicate samples and are representative of three independent experiments. B. Proliferation of HCT116 cells treated with varying doses of metformin for 72 h. Cell numbers are expressed relative to cell counts in control conditions (0 mM metformin). Each data point represents the mean ± SEM for triplicate samples. C. Nucleotide abundance of H1299 cells after 14 h of treatment with varying doses of metformin. Abundances were measured by LC-MS. D–E. OCR (D) and ECAR (E) of H1299 cells cultured for 6 h in the presence or absence of 5 mM metformin. The data represent the mean ± SEM for each condition (n = 6 samples per condition) and are representative of three independent experiments. F–G. Glucose consumption (F) and lactate production (G) of H1299 cells after 48 h of treatment with or without metformin (5 mM). The data represent the mean ± SEM for each condition (n = 3 samples per condition) and are representative of three independent experiments. *, p < 0.05; **, p < 0.01; ***, p < 0.001; p < 0.0001. Raw data for this figure can be found in S8 Data. (TIF) [file pbio.1002309.s014.tif]

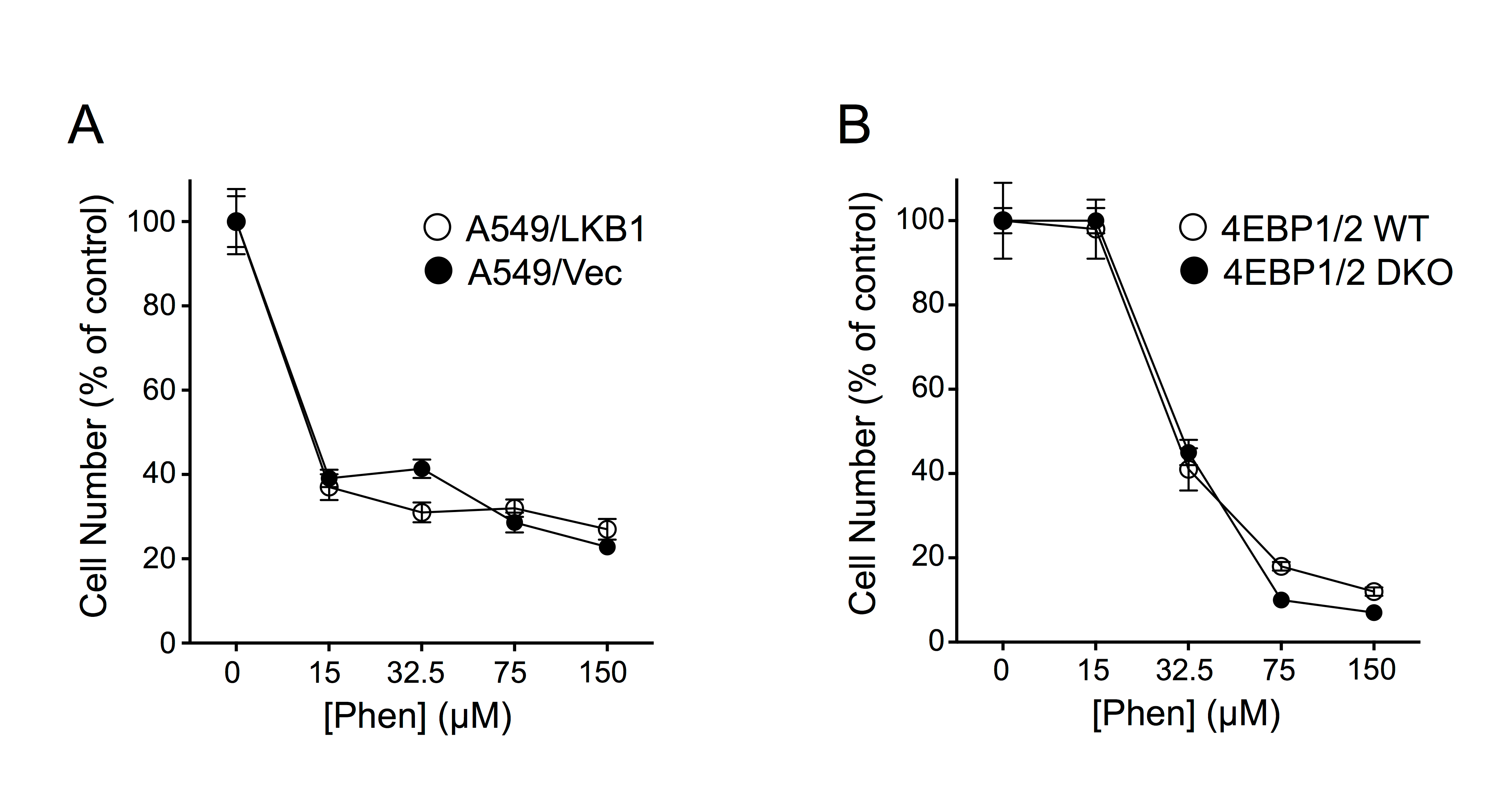

Supplement: S2 Fig — Related to Fig 2. A–B. Proliferation of A549 cells expressing empty vector (A549/Vec) or LKB1 vector (A549/LKB1) (A) and MEFs expressing WT 4EBP1/2 (4EBP1/2 WT) or knockout for 4EBP1/2 (4EBP1/2 double knockout [DKO]) (B), treated with varying doses of phenformin for 72 h. Cell numbers are expressed relative to cell counts in control conditions (0 μM phenformin). The data represent the mean ± SEM for each condition (n = 12 samples per condition), and are representative of two independent experiments. Raw data for this figure can be found in S9 Data. (TIF) [file pbio.1002309.s015.tif]

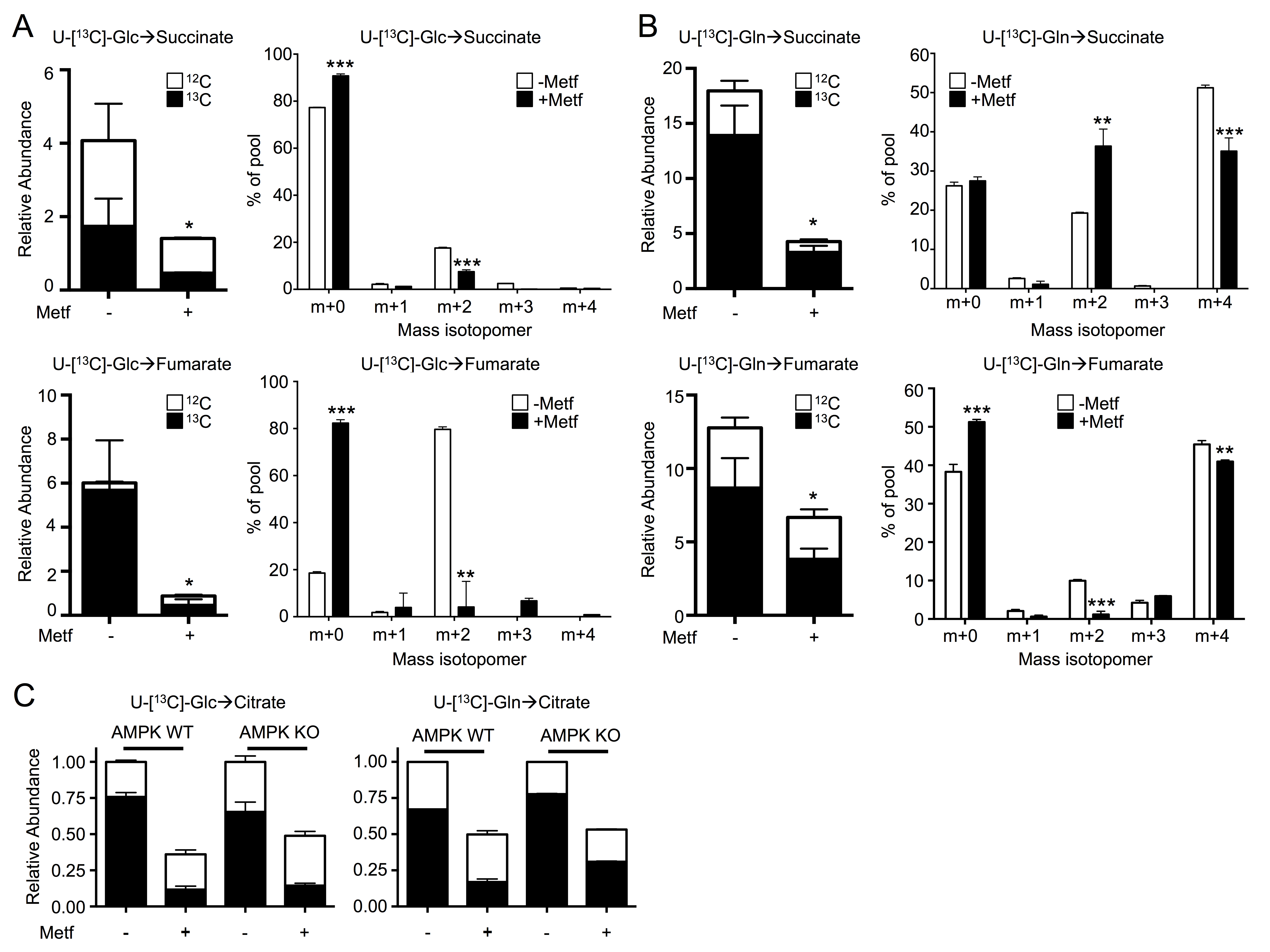

Supplement: S3 Fig — Related to Fig 3. A–B. H1299 cells were treated with (+) or without (−) 5 mM metformin for 6 h, followed by culture with U-[13C]-glucose (A) or U-[13C]-glutamine (B) for an additional 2 h. Shown is the relative abundance (left panel) and isotopomer distribution (right panel) for succinate (top panels) and fumarate (bottom panel) under each culture condition. Cells were then extracted and analyzed by GC-MS. C. Relative citrate abundance of MEFs, WT or KO for AMPK, after treatment with (+) or without (−) 5 mM metformin for 6 h, followed by a culture with U-[13C]-glucose or U-[13C]-glutamine for an additional 2 h. Data represents mean ± SEM for each condition (n = 3). Data shown is representative of three independent experiments. *, p < 0.05; **, p<0.01; ***, p<0.001; p<0.0001. Raw data for this figure can be found in S10 Data. (TIF) [file pbio.1002309.s016.tif]

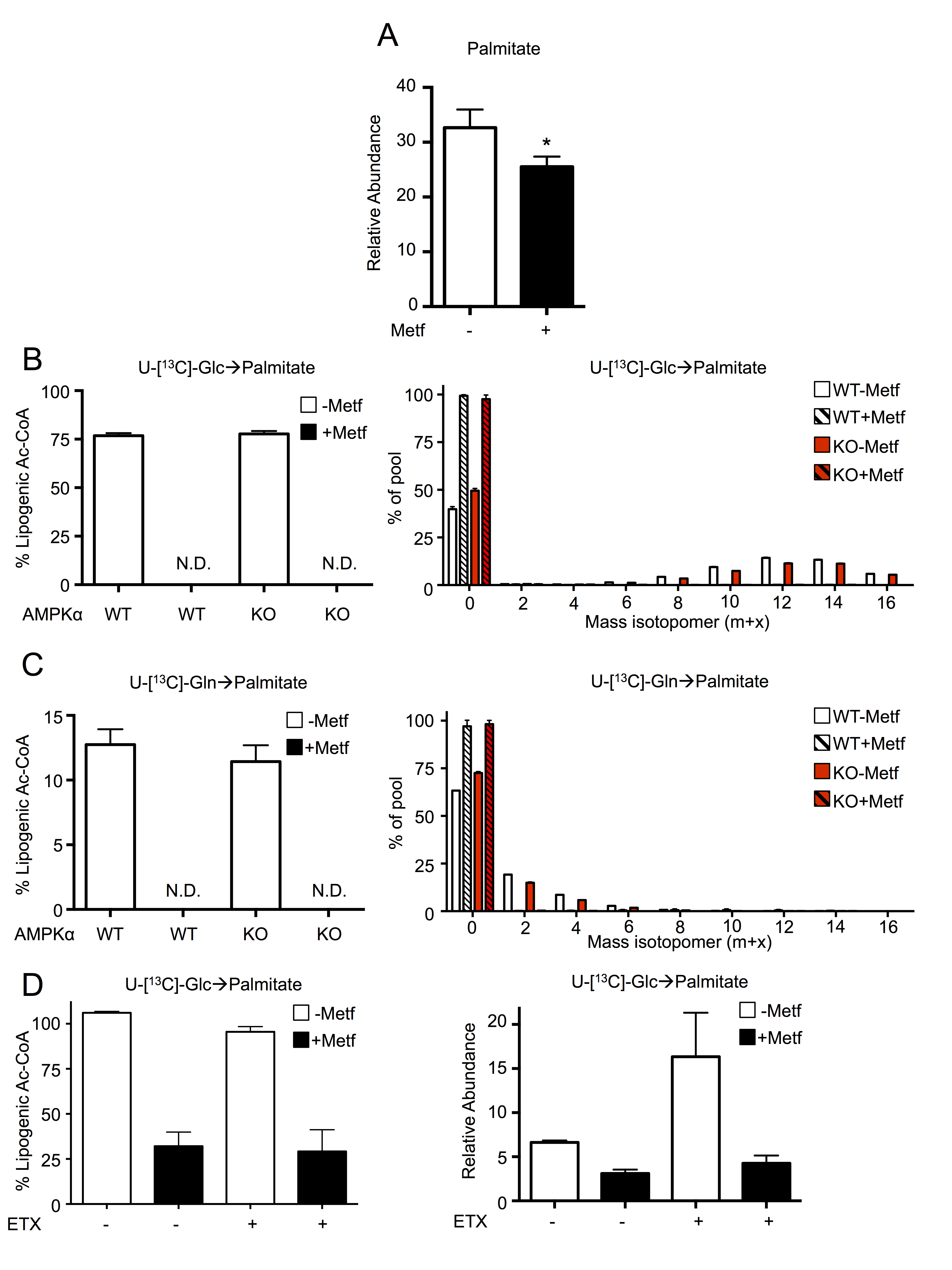

Supplement: S4 Fig — Related to Fig 4. A. Relative abundance of palmitate in H1299 cells following culture either in the presence or absence of metformin (5 mM) for 72 h. Cells were extracted following the exposure and analyzed using GC-MS. Data represent mean ± SEM for each condition (n = 3). Data shown is representative of three independent experiments. *, p < 0.05 B–C. Relative lipogenic acetyl-CoA and palmitate mass isotopomer distribution derived from U-[13C]-glucose (B) or U-[13C]-glutamine (C) in MEFs both WT (white) and KO (red) for AMPK after treatment for 48 h with (hashed bars) or without (open bars) 5 mM metformin and pulsed of 24 h. D. Relative lipogenic acetyl-CoA and palmitate abundance derived from U-[13C]-glucose in H1299 cells after treatment with (black bars) or without (white bars) 5 mM metformin for 48 h followed by glucose pulse with (+) or without (−) 200 uM etomoxir for 24 h. Data represent mean ± SD for each condition (n = 3). Data shown is representative of two independent experiments. Raw data for this figure can be found in S11 Data. (TIF) [file pbio.1002309.s017.tif]

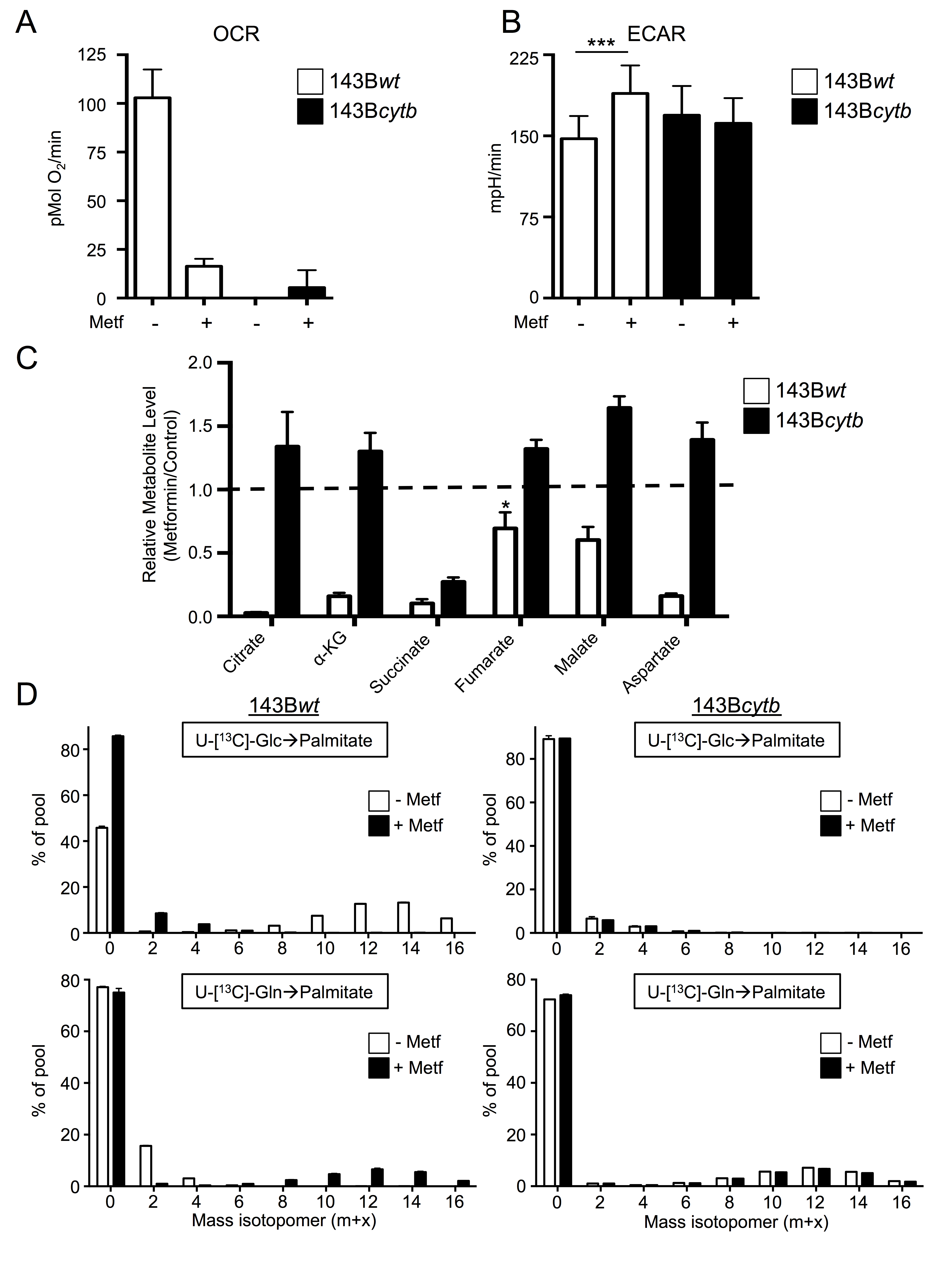

Supplement: S5 Fig — Related to Fig 5. A–B. OCR (A) and ECAR (B) of 143B osteosarcoma cells cultured with (+) or without (–) metformin (5 mM) for 6 h. C. Relative metabolite abundance of 143Bwt (white bars) and 143Bcytb (black bars) cells cultured with or without metformin (10 mM) for 12 h. Data expressed as a ratio of metformin treated cells over control conditions (0 mM metformin). Data represents mean ± SEM for each condition (n = 3). Data is representative of two independent experiments. D. Isotopomer distribution of U-[13C]-glucose- and U-[13C]-glutamine-derived palmitate of 143Bwt and 143Bcytb cells cultured with (black bars) or without (white bars) metformin (10mM) treatment. Cells were cultured either with or without 10mM metformin for 48 h followed by a 24 h pulse with either U-[13C]-glucose (top panel) or U-[13C]-glutamine (bottom panel). Data represents mean ± SEM for each condition (n = 3). Data shown is representative of three independent experiments. *, p < 0.05; ***, p < 0.001; p < 0.0001. Raw data for this figure can be found in S12 Data. (TIF) [file pbio.1002309.s018.tif]

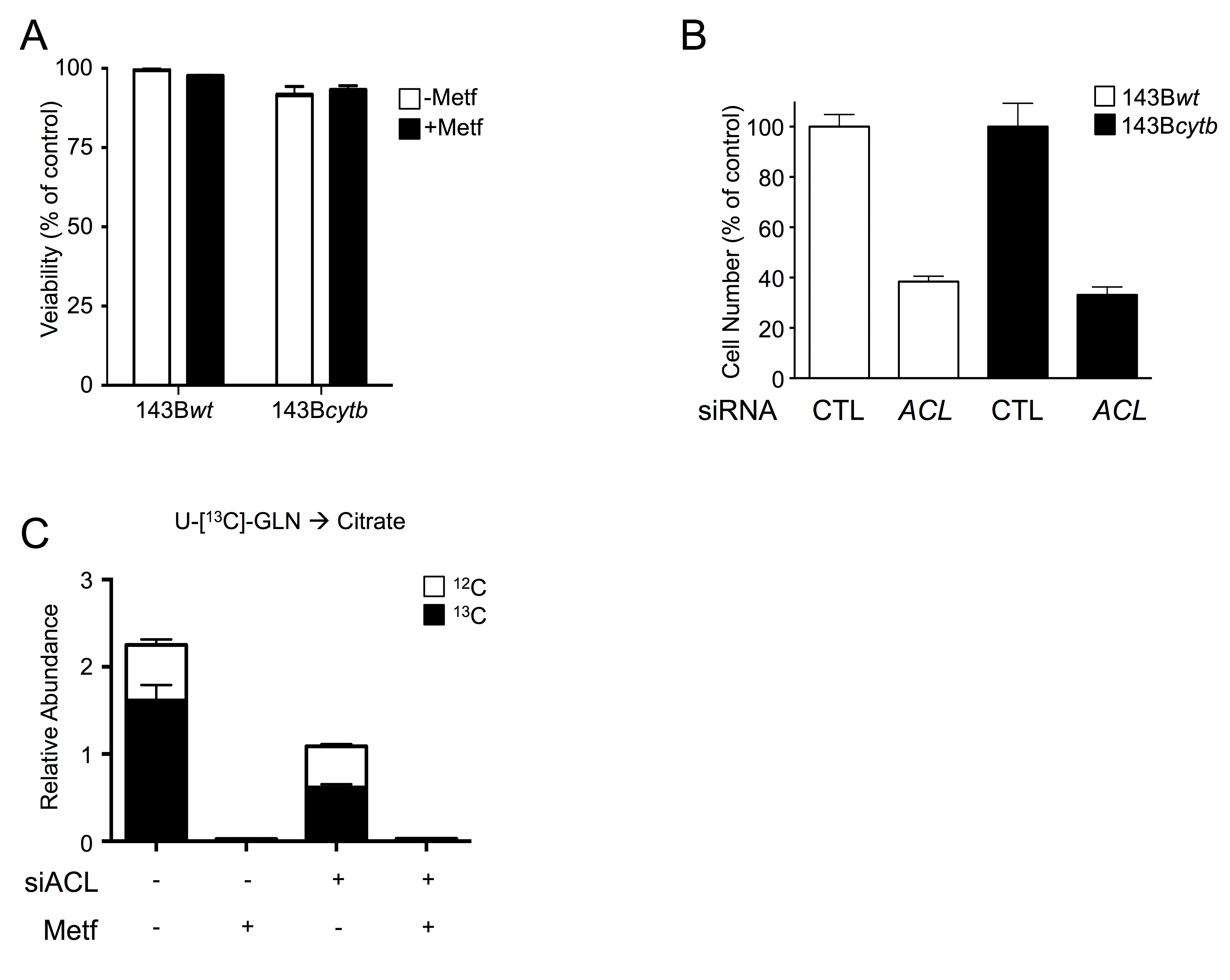

Supplement: S6 Fig — Related to Fig 6. A. 143B cell viability measured using propidium iodide incorporation. Cells were treated with (black bars) or without (white bars) 5 mM metformin for 72 h in regular growth media. Data normalized to control conditions. Data presented as mean ± SD for triplicate samples and are representative of three independent experiments. B. Proliferation of 143Bwt and 143Bcytb cells with control siRNA (CTL) or ACL siRNA (ACL). The data represent the mean ± SEM for each condition (n = 12 samples per condition), and are representative of two independent experiments. Cell numbers normalized to siRNA CTL conditions. C. Relative abundance of U-[13C]-glutamine-derived citrate in 143Bwt cells with (+) or without (−) 5 mM metformin with control siRNA (-) or ACL siRNA (+) treatment. Cells were treated with 5 mM metformin for 6 h followed by a glutamine pulse for 6 h. Data represents mean ± SEM for each condition (n = 3). Raw data for this figure can be found in S13 Data. (TIF) [file pbio.1002309.s019.tif]
